# The post-award effort of managing and reporting on funded research: a scoping review

**DOI:** 10.12688/f1000research.133263.2

**Published:** 2023-09-28

**Authors:** Ksenia Crane, Amanda Blatch-Jones, Kathryn Fackrell

**Affiliations:** 1 National Institute for Health and Care Research (NIHR) Coordinating Center, University of Southampton, School of Healthcare Enterprise and Innovation, Southampton, England, SO16 7NS, UK

**Keywords:** Post-award management, grant management, monitoring and reporting, compliance, assurance, research funding, research bureaucracy, research impact assessment

## Abstract

Introduction: Reporting is a mechanism for funding organisations to monitor and manage the progress, outputs, outcomes, and impacts of the research they fund. Inconsistent approaches to reporting and post-award management, and a growing demand for research information, can lead to perception of unnecessary administrative effort that impacts on decision-making and research activity. Identifying this effort, and what stakeholders see as unmet need for improvement, is crucial if funders and Higher Education Institutions (HEIs) are to streamline their practices and provide better support with reporting activities. In this review, we summarise the processes in post-award management, compare current practices, and explore the purpose of collecting information on funded research. We also identify areas where unnecessary effort is perceived and improvement is needed, using previously reported solutions to inform recommendations for funders and HEIs.

Methods: We conducted a scoping review of the relevant research and grey literature. Electronic searches of databases, and manual searches of journals and funder websites, resulted in inclusion of 52 records and 11 websites. Information on HEI and funder post-award management processes was extracted, catalogued, and summarised to inform discussion.

Results: Post-award management is a complex process that serves many purposes but requires considerable effort, particularly in the set up and reporting of research. Perceptions of unnecessary effort stem from inefficiencies in compliance, data management and reporting approaches, and there is evidence of needed improvement in mechanisms of administrative support, research impact assessment, monitoring, and evaluation. Solutions should focus on integrating digital systems to reduce duplication, streamlining reporting methods, and improving administrative resources in HEIs.

Conclusions: Funders and HEIs should work together to support a more efficient post-award management process. The value of research information, and how it is collected and used, can be improved by aligning practices and addressing the specific issues highlighted in this review.

**List of abbreviations**
CRIS-IR: Current Research Information Systems-Institutional Repositories
HEI: Higher Education Institution
ISRCTN: International Standard Randomised Controlled Trial Number
NIHR: National Institute for Health and Care Research
NJL: NIHR Journals Library
PRISMA-SR: Preferred Reporting Items for Systematic Reviews and Meta-Analyses (Scoping Reviews)
RA: Research administrator (see also RMA)
REF: Research Excellence Framework
RIA: Research impact assessment
RMA: Research management and administration
T&Cs: Terms and conditions

## Introduction

The availability of research funds is declining and is subject to tighter compliance, performance, and fiscal controls. ^1^ As funders are under more pressure to monitor and account for the research they fund, the effort of ‘post-award management’ is also increasing and affecting the productivity and culture in academic research. ^2^^–^^5^

Post-award management, also frequently known as ‘compliance’, ^6^ ‘grant management’, ^7^ and ‘monitoring and reporting’, ^8^ refers to the stages of research after a decision to fund has been made. These stages involve many tasks to set up and manage the research, including awardees reporting to funders on its progress, outputs, outcomes, and impacts. The effort involved (particularly for researchers) can be considerable, and administrative support is therefore important to avoid hindrance to research and spending too much time on grant-related administrative activity. ^9^ Where support is lacking however, too many compliance and reporting requirements can lead to faculty burden, ^9^ pressure on research careers and the training environment. ^10^ Despite this, practices in post-award management have been rarely explored by the research community, and resources related to funding and ‘grantsmanship’ remain focused on pre-award areas, such as applications and peer review. ^11^

The disruptive effect of the pandemic on research has led to renewed focus on academic support and removing administrative barriers to mechanisms of funding and research delivery. ^12^^,^^13^ In the United Kingdom, plans for reducing ‘research bureaucracy’ include making changes to practices that now crucially involve post-award management, and organisations are asked to streamline how they collect and share information to reduce duplication and delay in research. ^14^ While there is evidence that funders are indeed making changes to reduce administrative burden in practices, these still mostly focus on pre-award processes (e.g., shorter funding applications and contracts), ^15^^–^^17^ while post-award management remains an area of few visible changes. Funders and Higher Education Institutions (HEIs) must therefore show they are addressing their post-award processes and comparing their practices, involving other institutions and sponsors in making decisions (e.g., on methods of information collection), and explaining to researchers why information is asked for, and how it is used. ^14^

As strategic changes occur in this space, ^18^^–^^20^ they should be supported by better understanding of the tasks and effort that go into post-award management. The value of the process needs to be clarified to the research community, ^21^ and so should the roles that funders and HEIs play in supporting more efficient methods of research reporting. The aim of this review was to understand the current position and landscape of post-award management; catalogue and summarise the different activities involved; and explore the purpose of information collection in research. An additional aim was to compare how funders currently approach post-award management; identify any unnecessary effort or need for improvement; and to use evidence of previous solutions to inform recommendations for both funders and HEIs.

## Methods

The review followed the unpublished protocol of the authors and is based on the PRISMA-ScR and JBI methodological and reporting frameworks for scoping reviews. ^22^^,^^23^

### Eligibility criteria

We used a broad definition of ‘post-award management’ to capture any process relevant to the set-up, management, monitoring, or reporting of externally a funded research award (funds awarded to a HEI by a government agency or charity). Processes are relevant from when the decision to fund has been made (e.g., Notification of Award) and include any type of communication or request for information (e.g., progress report) from the funder or other external research stakeholder (e.g., government sponsor) to inform on the status, outputs, outcomes or impacts of research during award delivery. Relevant internal (HEI) processes include curation and management of research data and the financial management of awards. Other relevant funder processes include methods of collection and tracking of research data (e.g., for research impact assessments). Processes carried out during award close-out and post-completion are also considered ‘post-award’ and relevant, and include end-of-grant reports and tracking the long-term impacts of research.

To be included in the review, records had to be written in English, be accessible in full text or PDF format and describe any process(es) relevant to post-award management and reporting, as per the definition above. Records were excluded if they broadly referenced to post-award management without detail on the processes involved, or if they were out of the study scope (e.g., focusing on financial management of research awards or frameworks for research impact assessment). Eligible records could be peer-reviewed publications, grey literature (e.g., blogs, reports), presentations, or websites and no limit was placed on the publication date, status, or country to capture as much of the literature as possible in what we expected to be a sparsely explored area of research.

### Search strategy

All authors were involved in developing the electronic database search strategy used to identify relevant literature on post-award management and reporting of funded research.

To test the initial search term and keyword combinations, limited searches were conducted in Embase and Medline by the lead author (KC) to check the availability and relevance of titles and abstracts. Since combining all the search terms returned no results in either database, the search strategy had to be refined, whereby multiple separate single-term searches were conducted ( Table 1) and the results of each search were screened separately. This process was followed by searches using search term/keyword combinations to narrow searches where possible (as shown in Table 1) and these results were also screened.

**Table 1.** Example of the search strategy used to identify relevant records in databases. Searches are shown in the order they were conducted, and each line represents a separate search, the results of which were separately screened.

Search terms	Electronic databases
‘post-award management’ OR ‘post award management’ (title and abstract) ‘post-award’ OR ‘post award’ ‘research manage*’ (limited to abstracts) ‘grants process’ OR ‘grant management’ (limited to abstracts) ‘grant reporting’ ‘research contract*’ OR ‘research contracting’ OR ‘research contracting process’ ‘research progress report’ OR ‘progress reporting’ ‘research impact assessment’ ‘Researchfish’ ‘research impact’ (limited to abstract) ‘bureaucra*’ AND ‘research’ ‘monitoring’ AND ‘research’ AND ‘funder’ (Filters: English language, full-text)	Web of Science, Medline, Embase, PubMed

Full literature searches were undertaken in relevant electronic databases, namely Embase, Medline, Pubmed, Web of Science and Google Scholar. Additionally, manual searches of the content tables of key journals (determined using the interquartile rule for outliers) were conducted to find relevant articles published in the last year (2022-23). Initial searches were conducted in March 2022 and the final manual searches were conducted in March 2023.

In addition to electronic and manual searches, the websites of 11 funding organisations (listed in Table 2) were reviewed to obtain information on current funder approaches to post-award management. The funders (listed in Table 2) were chosen to incorporate a range of geographical regions, funder sizes and monitoring and reporting approaches. The websites were reviewed between March and October 2022, with updated review in February 2023. The dates the websites were accessed and links to the web pages searched are shown in Table 2.

**Table 2.** Details of funder websites searched. Relevant web links include funders’ homepages and pages dedicated to award management, monitoring, and reporting. Where available, links to reporting content (including downloadable templates) are also included.

Funder	Relevant web links	Dates accessed
Alzheimer’s Research UK	https://www.alzheimersresearchuk.org/ https://www.alzheimersresearchuk.org/research/for-researchers/measuring-impact/researchfish-faq/	1 April 2022 and 2 February 2023
Canadian Institutes of Health Research	https://cihr-irsc.gc.ca/e/193.html https://cihr-irsc.gc.ca/e/45317.html https://cihr-irsc.gc.ca/e/40176.html	15 June 2022 and 3 February 2023
European Research Council	https://erc.europa.eu/homepage https://erc.europa.eu/manage-your-project/scientific-reporting	18 April 2022 and 3 February 2023
Health Research Council New Zealand	https://www.hrc.govt.nz/ https://www.hrc.govt.nz/resources/data-monitoring-core-committee https://gateway.hrc.govt.nz/	1 July 2022 and 12 January 2023
Medical Research Council	https://www.ukri.org/councils/mrc/ https://mrc.ukri.org/funding/guidance-for-mrc-award-holders/ https://www.ukri.org/wp-content/uploads/2021/02/UKRI-020221-Additional-Funders-Questions-Overview.pdf https://www.ukri.org/what-we-offer/what-we-have-funded/research-and-innovation-outputs/ https://www.ukri.org/about-us/mrc/performance-monitoring-and-evaluation/	23 March 2022 and 2 February 2023
National Health and Medical Research Council	https://www.nhmrc.gov.au/ https://www.nhmrc.gov.au/funding/manage-your-funding/reporting https://www.nhmrc.gov.au/funding/manage-your-funding/reporting/progress-final-and-additional-reporting#download https://healthandmedicalresearch.gov.au/tutorials.html	15 June 2022 and 3 February 2023
National Institute for Health and Care Research (NIHR)	https://www.nihr.ac.uk/ https://www.journalslibrary.nihr.ac.uk/information-for-authors/ https://www.journalslibrary.nihr.ac.uk/about-us https://www.nihr.ac.uk/search-results.htm?search=draft+final+report https://www.journalslibrary.nihr.ac.uk/information-for-authors/publication-types/Threaded-Publication/index https://www.nihr.ac.uk/about-us/who-we-are/our-policies-and-guidelines/ https://www.hra.nhs.uk/planning-and-improving-research/policies-standards-legislation/research-transparency/make-it-public-transparency-and-openness-health-and-social-care-research/#reporting	1 April 2022 and 2 February 2023
National Institutes of Health	https://www.nih.gov/ https://grants.nih.gov/grants/post-award-monitoring-and-reporting.htm https://grants.nih.gov/grants/rppr/index.htm ( https://report.nih.gov/about) https://grants.nih.gov/policy/index.htm#gps	18 April 2022 and 3 February 2023
National Research Foundation Singapore	https://www.nrf.gov.sg/ https://researchgrant.gov.sg/Pages/TrainingGuides.aspx	9 October 2022 and 3 February 2023
University Grants Committee Hong Kong	https://www.ugc.edu.hk/eng/ugc/index.html https://www.ugc.edu.hk/eng/rgc/guidelines/Governing/report_forms.html	1 July 2022 and 13 January 2023
Wellcome	https://wellcome.org/ https://wellcome.org/grant-funding/guidance/end-grant-reporting https://wellcome.org/grant-funding/guidance/wellcome-clinical-trial-policy-monitoring-2018-2022#why-we're-monitoring-compliance-4243	23 March 2022 and 2 February 2023

### Study selection process

Records identified through database and manual searches were exported with citation, titles, and abstracts into Endnote 20 (Clarivate, UK). Duplicates were removed using the EndNote duplication function and manually. Records were divided into two groups to screen titles and abstracts against the eligibility criteria. Each group was independently screened by two authors (AJBJ and KF), with the lead author (KC) screening all records. Any disagreements between authors regarding the decision to include or exclude were resolved through discussion until consensus was reached. Full texts were retrieved for all records that were agreed to be included at this stage. Where full texts could not be retrieved, access to full texts was requested from the University of Southampton Library Services. Two authors (KC and AJBJ) screened all the full texts of records, and if agreement to include or exclude was not reached the third author (KF) was consulted to arbitrate.

### Extracting, cataloguing, and summarising data

A structured data extraction form was created in Microsoft Excel and piloted using five eligible records. Following team discussions, the form was revised before formal extraction began. The lead author extracted the data for each record. Extraction fields (shown in Box 1) included: (i) study identifiers, (ii) study characteristics, (iii) details on post-award management processes, (iv) descriptions of unnecessary effort or need for improvement, and (v) descriptions of any previous solutions or recommendations from authors.

Box 1. **Data extraction fields for records and funder websites.**

HEI=Higher Education Institution.

**Table:** 

Data extraction fields for records:	Data extraction fields for funders websites:
Author(s) Date of publication Title/abstract, Publication type Lead author affiliation Topic and aim Study design (if research) Research funding field Organisational level (HEI or funder) Award type(s) Terminology used for post-award management (e.g., ‘monitoring’) List of specific processes, requirements, and tasks Justifications for use of specific processes or needing post-award management in general Digital systems Stakeholders involved (e.g., the principal investigator) Descriptions of unnecessary effort or need for improvement. Descriptions of previous solutions (e.g., interventions, pilots, and training) Recommendations from authors	Funder name Year of latest update (relevant pages) Terminology used for post-award management (e.g., ‘monitoring’) Digital systems Award type(s) Whether the same monitoring and reporting requirements are applied to all awards/schemes (Y/N) List of specific processes, requirements, and tasks used for the main programmes/awards, Frequency of progress reporting (e.g., annual) Summary of the report content (if available) Information on who in HEIs is responsible for fulfilling requirements (e.g., project director) Guidance for researchers and HEIs Justifications for use of specific processes or the need for post-award management in general Information on relevant policies (e.g., monitoring and evaluation) Links to relevant pages

An additional data extraction form was created for information on the current post-award management practices of funders, as obtained from their websites. The lead author extracted data from 11 websites in total, with the relevance of all information checked by all authors. The extraction fields are shown in Box 1.

Extracted information on specific processes, requirements and tasks was analysed thematically, whereby similar processes were categorised and the resulting categories represented the main components of the post-award management processes. Each category was then ordered according to its frequency in the literature (i.e., the number of citing records) to give an idea of the effort involved. In adaptation of a method used by Glonti et al. to explore the roles and tasks of journal reviewers, ^24^ extracted information relating to the purpose of post-award management was collated and used to compose common purpose-related statements ( *“The purpose of post-award management is …”*). To do this, all relevant information was extracted into a Microsoft Excel spreadsheet by KC. Subsequently, information was coded into common statements and similar statements were categorised into overarching themes and ordered by frequency, again based on the number of citing records. Finally, extracted information relating to unnecessary effort or need for improvement in post-award management was summarised for each publication and thematically categorised according to the component of post-award management (e.g., research impact assessment) the information was relevant to. This information, along with any information on previous solutions or authors’ recommendations, was used to inform broad recommendations for funders and HEIs.

The information obtained from funder websites was extracted into and collated in Microsoft Excel and used to draw out the main points of variation in how funders approach post-award management. This included comparison of reporting requirements and the frequency of reporting, use of digital systems for award management, funder policies, resources and evidence of support.

## Results

### Selection of records of evidence

**Figure 1.**
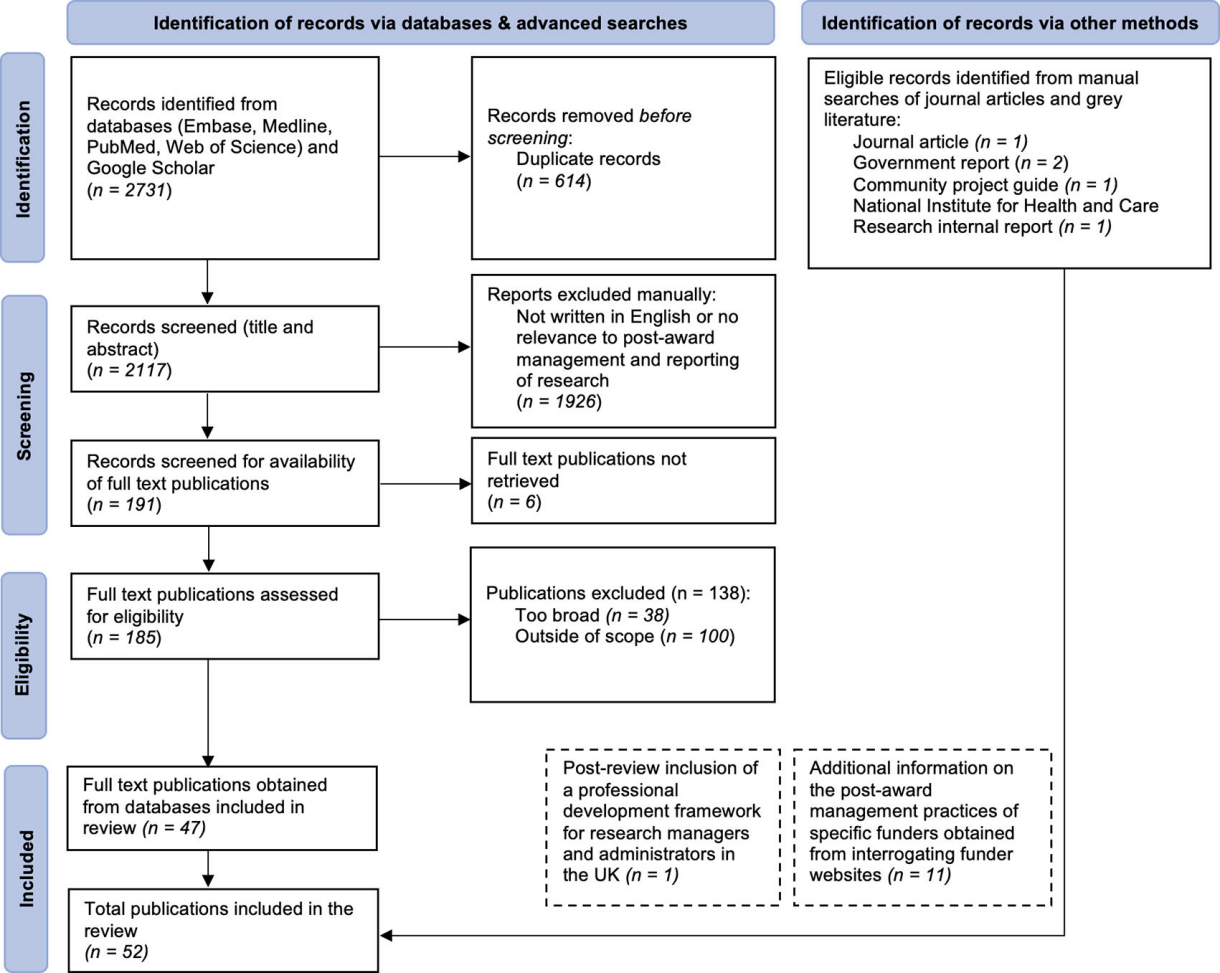
PRISMA flowchart of the search strategy used to identify records and relevant web information on the post-award management and reporting of funded research.

The search strategy is depicted in Figure 1. Database searches yielded 2731 eligible records. Duplicate records were removed, and of the remaining 2117 records, 1926 records were excluded through title and abstract screening for not having met our eligibility criteria. Following full text screening of the remaining 191 records, a further 144 records were excluded (due to being too broad (n=38), out of the scope (n=100), or the full texts could not be retrieved (n=6)), which left 47 records to be included in data extraction. Manual searches identified a further 4 eligible records, giving a total of 51 records that were ultimately included in the review. Following peer review, we also included an additional record: a professional development framework for research managers and administrators in the UK ( Table 3), bringing the total number of records to 52.

**Table 3.** Summary of findings from records on post-award management (n=52). Asterisk ( ^*^) denotes an unpublished report obtained with permission from the National Institute for Health and Care Research (NIHR) Coordinating Centre.

Author(s)/year	Publication type	Funding field (and country)	Extracted information on post-award management processes, information, and reporting requirements			
			Organisational level (Higher Education Institution (HEI) or Funder)	List of processes, tasks, or requirements described (must have)	Systems used to support processes (if any)	Stakeholders mentioned in the process (if any)
Abdullahi et al., 2021 ^44^	Mixed-methods study	Small grants in health research (Kenya)	Funder	Promotion of funded research on digital and non-digital platforms, reporting collaborations, feedback, and recommendations for program improvement	Not specified	Principal investigators
Abudu et al., 2022 ^58^	Review	Research grants and contracts	Funder	Annual or end-of grant reports, reporting research outputs (citations/publications, research accomplishments, collaborations/networks, capacity-building, career advancement, future funding, research targeting, media citations/presentations) and outcomes (products/research tools, patents, drugs, clinical practice policy/commission memberships), and impacts (broader health economic or societal downstream impacts of research, or ROI studies), administrative or financial data related to projects, Researchfish reports, surveys or semi-structured interviews with investigators	Dimensions.ai, Researchfish	Principal investigator or grantees, contracting officer or project representative
Adam et al., 2018 ^36^	Opinion piece	Health research contracts/grants (Spain)	Funder	Reporting on research impact, plain English summary in all reports	Not specified	Not specified
Adam et al., 2012 ^35^	Case study	Extramural grants in clinical and health services research (Spain)	Funder	Progress reports on achievements, results, and changes to work plan, declaration of all outputs	Not specified	Principal investigators
Agostinho et al., 2020 ^79^	Case study	Research and Innovation (Portugal & Spain)	Funder and HEI	Contract negotiation, adherence to funder and statutory Ts & Cs, compliance with auditing requirements	Not specified	Institutional research support staff
Aliyu et al., 2021 ^80^	Evaluation	Clinical and health research (USA & Nigeria)	HEI	Annual performance report, research performance progress report, financial tracking and reporting, subcontract management and compliance, fiscal oversight, effort reporting	Research Electronic Data Capture protocol tracking database (REDCap)	Office of Research Administration (grant managers, project coordinators, fiscal accounting staff), Institutional Review Board, Ethics Review Board, Community Advisory Board
Al Mawali et al., 2016 ^81^	Review	Health research (Oman)	HEI and funder	Progress reports, final reports, clinical trial registration	Ministry of Health Centre for Studies and Research (MoHCSR) website	Not specified
Allen, 2016 ^37^	Blog	Research (UK)	Funder	Reporting of grant outputs, products, and impacts (using common taxonomies developed by funders and policy makers)	ResearchFish, Pure, Converis, ImpactTracker	Not specified
Association of Research Managers and Administrators, 2011 ^25^	Professional Development Framework	Research (UK)	HEI and funder	Drafting, negotiating, and accepting contracts, dealing with project finance, employing staff on research contracts, reporting to funders, collecting data on research outputs and supporting researchers to collect evidence of research impact, promoting knowledge exchange, supporting technology transfer, providing sound administrative support (for researchers and students), contributing to organisational and funder policies (e.g., Open Access), contributing to the Research Excellence Framework, supporting research ethics and governance, working with information systems, supporting audit, and making statutory returns.	Research Excellence Framework (REF)	Research managers and administrators (RMA), postgraduate researchers, funders, research staff
Bagambe, 2012 ^67^	Journal abstract	Biomedical research grants (Uganda)	HEI and funder	Grants acquisition and management (from funding acquisition to project closure), contract negotiation, programmatic and financial compliance with funder requirements, monitoring and evaluation	An automated System for Integrated Grants Management (SIGMA)	Institutional and sponsor grant managers
Baghurst, 2021 ^19^	Commissioned report	Health and social care research (UK)	Funder	Open Access policy compliance, plain English summary, reporting publication DOIs, reporting employer and funder affiliations, acknowledgements, ORCID ID numbers	CRIS, Researchfish,	Researchers, research managers, library services
Basner et al., 2013 ^77^	Prospective evaluation	Federal clinical research (USA)	Funder	Semi-annual progress reports (overall center progress, research projects, cores, education and training unit, outreach dissemination unit, collaborations, in-progress publications, peer-reviewed publications, leveraged funds, patents, trainees, courses, eetings and outreach activities)	Interdisciplinary Team Reporting Analysis and Query Resource (iTAQR), NIH Research Portfolio Online Reporting Tools and Expenditures and Results) RePORTER	Investigators, trainees
Bates & Jones, 2012 ^48^	Guide	Public health and community research (UK)	Funder and HEI	Periodic progress reporting: inputs, day-to-day activities, outputs, and outcomes (e.g., tracking types of events, activities, costs of delivery and characteristics of users), dissemination within community (e.g., social media), reporting of findings for external/internal feedback, publication of articles	Not specified	Project participants, sponsors, commissioners, volunteers
Bhurke, Chinnery & Raftery, 2018 ^*^^52^	Report	Health and care research (UK)	Funder	Grant acceptance and special conditions, compliance with T&Cs, final report, progress report, mid-term report, annual report, invoicing, extension request, publication	Not specified	PI/CI, host institution, funder, administrator, officer, programme manager, research advisor, head of research funding, director, business development team, trial steering committee
Bird, 1995 ^82^	Book chapter	Ethics in research	Funder	Intellectual property, authorship, individual contributions, data retention policy, publication, progress milestones, notification of approval for changes (e.g., to research design or objectives)	Not specified	Not specified
Bonham & Barnes, 2020 ^83^	Journal feature	Extramural grants in health research (USA)	Funder	Reporting ‘foreign influence’ such as: financial conflict of interest (including travel to affiliated institutions), foreign or personal funding, ‘time commitment’ to foreign institutions, conflicting IP’s or authorship/expected co-authorship, internal review of grant holders and projects, and reporting corrective actions to agency (in the case of foreign inquiries)	Not specified	Principal investigators, co-investigators, agency managers, government regulators, law enforcement and US Congress
Briar-Lawson et al., 2008 ^84^	Case scenario	Federal social research (USA)	HEI and funder	Expenditure reports, purchasing of materials, hiring staff, travel arrangements, payments to research participants	Not specified	Principal investigator, HEI administrators, business managers, Associate Dean of research
Brouard and Glass, 2017 ^85^	Conceptual article	Philanthropic foundation research (Canada)	Funder	Descriptive reporting of activities and outcomes, including end of project reports, testimonials and success stories, external evaluations of project/program, audited financial statements. Site visits, presentation to foundation board. Evaluation and ‘performance measurement’ of outcomes of activities.	Not specified	Principal investigators, grant managers and foundation staff/board
Buck, 2014 ^38^	Correspondence	Research (UK)	Funder and HEI	Annual output, outcomes, and impact reporting	Researchfish	Not specified
Burland and Grout, 2016 ^39^	Journal abstract	Research (UK)	Funder and HEI	Data management and compliance with policies of open access, accessibility/discoverability, and research administration standards. Reporting of outputs and impacts of research	Jisc, Current Research information Systems (CRIS/IR), CASRAI, ISNI, Je-S, Gateway to Research (GtR), open-source platforms, repositories, databases/spreadsheets	Not specified
Clements et al., 2017 ^86^	Journal abstract	Research (UK)	HEI	Reporting publication output, intellectual property (IP) and engagement activities, acknowledging funding source in publications	Researchfish, Current Research Information System (CRIS/IR)	Investigators, programme managers
Collado et al., 2017 ^8^	Mixed-methods pilot study	Philanthropic health research (UK)	Funder	Reporting impact (narrative via telephone interviews): outputs, grant products, direct grantee outreach, impact activities	Researchfish	Principal investigators, designated grant monitor
Corona Villalobos, 2020 ^29^	Thesis	Administration research (USA)	HEI	Research administrator tasks: serving as a point of contact for federal grant managers, ensuring compliance (e.g., ethics, health and safety), implementing and reporting on federal grants, coordinating site visits or reviews, coordinating internal/external audits, completing financial and programmatic progress reports, grant closures, facilitating training	Not specified	Research administrators, Principal investigators, federal grant managers
Croxson, Hanney & Buxton, 2001 ^49^	Journal article	Health-related R&D (UK)	Funder	Reporting measurable outputs: publications, patents granted, higher degrees awarded, conferences/meetings, references in published policy reports & guidelines, annual reports, self-reporting (e.g., questionnaires)	UK Economic and Social Research Council REGARD system, Research Outputs Database (Wellcome)	Not specified
Davidson et al., 2014 ^40^	Pilot	Research (UK)	HEI	Compliance with data management and sharing policies, Data Management Plan, data registration	Researchfish, Digital Curation Centre Data Management Plan (DMP) online tool, CRIS, Edinburgh DataShare, Research data registry and discovery service (RDRDS), Jisc	Researchers, data managers, funders
Decker et al., 2007 ^7^	Federal report	Federal research (USA)	Funder and HEI	Periodic scientific progress reports, financial reports, certifying the effort of research participants	Not specified	Principal investigators and co-investigators, administrative faculty staff
DeMoss et al., 2018 ^6^	Journal article	Research (USA)	Funder and HEI	Review of award notice Ts & Cs, establishing accounts and allocating budgets, providing account information, effort reporting, setting up subcontracts, monthly account reconciliation, individual project and overall portfolio analysis, process effort changes, annual financial reports, forecasting/burn rates, review and transfer of trailing charges, project inactivation, Open Access policy compliance, financial conflict of interest	Not specified	Post-award accountants
Dresen, 2012 ^30^	Exploratory survey and usability study	Federal research (USA)	Funder and HEI	Monitoring grant outcomes, budget, compliance with HEI and federal requirements, time and effort reporting, grant budgeting and accounting, financial conflict of interest, institutional IRB protocol, expenditure for contracted services	Not specified	Faculty research staff and students, sponsored program administrator, research administrators
Flores-Rivera, 2020 ^31^	Implementation of a faculty services model	Research (USA)	Funder and HEI	Progress reports, subcontract-related invoicing, accounts payable, monitoring award balances, budget forecasting, record preparation and review, processing personnel, purchasing transactions, budget oversight and proposal processing, signoff, compliance, and data security, hiring, Just-In-Time requests, award-set up and financial support, submission of requests to pre- and post-award offices (e.g., for extensions, revisions), effort reporting, cost sharing transfers	New Oracle Cloud HCM and Finance Platforms	Research administrators, faculty staff, researchers
Fowler & Zitske, 2015 ^87^	Presentation	Agricultural and life science research (USA)	HEI	Compliance with grant Ts & Cs, progress reporting, purchase documentations, technical and fiscal closeout, and beyond award activities (record retention, property control, audits)	Not specified	Principal investigators, faculty staff, dean’s offices, accounting and business services, Research and Sponsored Programs office (RSP)
Guthrie et al., 2019 ^47^	Report	Health and medical research (Australia)	Funder	Grantee variation requests (e.g., change in research plan, contracted investigator, supervisor, institution, FTE, commencement date), financial acquittals, end of grant reporting (plain English summary, research achievements, likely impact on health, economy, community, policy, updated CV and featured outputs linked to grant ID), information on commercialisation (patents, spin outs, income from IP, licence agreements, industry co-funding), innovation (collaboration, other disciplines involved, journals cited), development and implementation of novel interventions, tools, therapies, processes or services	Sapphire grant management system	Principal investigators
Hinrichs, Montague & Grant, 2015 ^34^	Report	Research grants & contracts (UK)	Funder	Recording research outputs, outcomes, and impacts	Researchfish	Researchers, evaluators
Hunter et al., 2014 ^33^	Review	Environmental and global health (UK)	HEI and Funder	Financial reporting, ethical clearance, governance	Not specified	Grantees and students, HEI administrators, funder consortium
Kane et al., 2021 ^88^	Observational study	Clinical research (USA)	Funder	Annual research performance progress report (or quarterly, if behind targets), enrolment data	Not specified	Federal program officers
Kasim et al., 2021 ^32^	Case study	Science research (Malaysia)	Funder	Research project performance, financial project report, presentation of outcomes and results after project completion	Not specified	Centre for Research Grant Management, researchers
Knowles et al., 2020 ^28^	Retrospective study	Clinical research (UK)	Funder	Annual compliance monitoring, after end-of-grant reporting of results summary (public), annual output reporting, registration of clinical trials with public trial registries, informing funder of trial registry number	Researchfish, Gateway to Research, trial registries (e.g., ISRCTN, EUCTR, clinicaltrials.gov)	Principal investigators, grant managers, regulators, and policymakers (UK House of Commons Science and Technology Committee)
Magee, 2010 ^89^	Conference abstract	Translational research (USA)	Funder	Registration of clinical trials with public trial registries (e.g., clinicaltrials.gov), budget compliance (and post-award revisions), staff expenses and payments, invoicing, enrolment data, adherence to study protocol	Online clinical trial registries (e.g., clinicaltrials.gov)	Principal investigators, co-investigators, clinical research managers/administrators
McLaurin & Gray, 2020 ^90^	Presentation	Research grants (USA)	HEI	Award and account set up, submission of reports, requests for no-cost extensions, financial management of award, changes in scope of work/key personnel/Ts& Cs/budget/planned or existing subawards	Not specified	Institutional research administrators, advisors, grants and contracts specialists, sub-award analysts
Muller, 2009 ^45^	Review	Federal research (USA)	Funder	Reporting, informal discussions and site visits, grantee-funder engagement, closeout (final reports, financial accounting and reconciliation)	Not specified	Grantee, sponsoring agency
Nature, 2021 ^50^	Commentary article	Research (UK and USA)	Funder	Journal outputs and research income	Not specified	Principal investigators and research managers
Norris, 2006 ^91^	Mixed-methods case study	Arts research (USA)	Funder	Final report: final budget, project activities, community involvement/participation, programs (e.g., new programs), administration (e.g., new staff), partnerships/collaborations, publications)	Not specified	Grantees
Lehman, 2016 ^42^	Exploratory sequential mixed-methods study	Research (USA)	HEI	Accounting, accounts payable and receivable, property and inventory control, payroll, and reporting	Electronic Research Administration (eRA) system	Investigator, research administrators, HEI leadership, IT support, sponsoring agency
Riechhardt, 1998 ^51^	Nature news article	Federal research (USA)	Funder and HEI	Institutional grant approvals	Not specified	Grant and top managers at sponsoring agency, government officials (Office of the Inspector General)
Roudebush & Moore, 2018 ^75^	Book chapter	Health research grants (USA)	Funder	Compliance with reporting requirements in Notice of Award, progress and final reports (project milestones, personnel changes, analyses/results, unexpected events, enrolment reports, popular media contacts/reports, presentations or manuscripts, preliminary results, What’s Next), financial report (summary narrative and how the money was spent)	Electronic Research Administration (eRA) Commons status page	Principal investigator, program officer, foundation grants manager, funding agency budget manager
Sajdyk et al., 2014 ^46^	Conference abstract	Federal research (USA)	Funder	Progress reporting: barriers to research, key outcomes, feedback on programme (survey)	REDCap (integration software system for data collection)	Programme managers, principal investigators
Sakraida et al., 2010 ^92^	Journal article	Federal health and behavioural research (USA)	Funder and HEI	Compliance with federal guidelines, funding agency standards and patient safety. Participant recruitment and retention procedures, follow-up telephone calls, preparation of information packets, manuscript writing, set up and maintenance of project office, data management and analysis procedures, dissemination of study outcomes, liaising with media/publicity, authorship and acknowledgement of research and support staff and students, progress reports, grant budget management and expenditure recording		Research administrative staff/project office, principal investigators, students, project director, oversight and compliance officers, grants and contracts officer, IRB, publicity/media consultants
Scacchi et al., 1997 ^93^	Article	Naval research (USA)	HEI	Funds expenditures, grant renewals, no-cost funds extension, invoicing and funds transfers, in-progress procurement actions, regulatory compliance, record keeping practices, and resource control, tracking reports resulting from research grant awards	INRIS, CAMIS, STARS	Office of Naval Research, researchers
Sundjaja, 2019 ^43^	Mixed-methods study	Research grants (Indonesia)	HEI	Monitoring and evaluation of program implementation, evaluation of grant requirements, reporting research activities, revising grant strategy, budgeting, data accessibility, relationship management	Review of systems (Good Done Grant Management System, JK Group Easymatch, SmartSimple GMS360, Npower Foundation Connect, Fluxx, Bromelkamp, Akoya, Versale Grants, MicroEdge GIFTS Alta)	Grant administrator, grantee
Tan et al., 2021 ^94^	Computational modelling	Research (Singapore)	HEI	Reporting milestones and deliverables, expenditure and justifications, publications, and IP	Project Reporting Management System (PRMS)	Principal investigators, higher management
Thomas et al., 2019 ^95^	Report	Health and care research (UK)	Funder	Responding to government requests for evidence, reviews, and inquiries, serving on advisory committees, participating in working groups/consultations, reporting patient and public involvement, collaborations within/outside of UK, publications, consultancy reports, policy briefings, post-award employment	Researchfish	Award holders
Tickell, 2022 ^14^	Interim report	Research (UK)	Funder and HEI	Reporting on project finances, data management, due diligence, export control, sponsorship for health and social care research, animal testing licenses, bullying and harassment, institutional concordats and strategic frameworks, budget change requests, extension requests	Flexi-grant system, Worktribe, UKRI Funding Service	Researchers, funders, HEIs
Tickell, 2022 ^14^	Final Report	Research (UK)	Funder and HEI	As above (Tickell, 2022 interim report), including post-award set up of finances, procurement and recruitment, project change requests, no-cost extensions, collaboration agreements, internal approvals, data management, reporting	UKRI Funding Service, Jisc	Researchers, institutional staff, government, regulators, funders
Viney, 2013 ^18^	Presentation	Health research (UK)	Funder	Reporting outputs, outcomes, and impacts	Researchfish	Not specified

### Characterising the literature

Just over half of the records on post-award management and reporting consisted of primary research studies and reviews (27 records; 51%). Research included mixed-methods studies, observational studies, case studies, evaluations (prospective and retrospective), needs assessments and computational models. The remaining 25 records (48%) were non-research (e.g., opinion pieces and blogs) or included reports, results of pilots (e.g., of funding programmes and grant management systems) and a professional development framework for research managers and administrators. Where research fields were specified, most were related to health and clinical research (19; 37%), with less represented fields including agricultural and life sciences (2; 4%), social research (1; 2%), the arts (1; 2%) and the study of research administration (1; 2%). The remaining records were deemed widely generalisable, as they were not aimed at any specific research field or type of research funding (24; 46%). Records were predominantly from the USA (21; 40%) and the United Kingdom (18; 35%), followed by Europe (3; 6%), Africa (3; 6%), South Asia (3; 6%) and the Middle East (1; 2%).

All records described processes relevant to post-award management; however, there was significant variation in the terminology used by authors ( Box 2), with most frequent terms including ‘grant management’, ‘monitoring’, and ‘monitoring and reporting’. In terms of the organisational level, half of the records (26; 50%) focused ‘externally’ and described the tasks and information requirements (e.g., progress reports) that funders or other sponsors external to HEIs request on funded awards. In contrast, only 10 records (19%) focussed on the HEI side of the process by describing the administrative and technical operations involved in the internal set up of awards (e.g., financial approvals, staff hiring) and the tasks related to compliance and assurance reporting (e.g., data management, preparing audit reports). In almost a third of the records (16; 30%), authors explored both the funder and HEI sides of the process, describing mechanisms relevant to both.

Box 2. **List of terminologies others use when referring to post-award management, as captured form the literature and funder websites.**

Square brackets ([]) indicate the possible variations in terms.

Administration of awards

Assurance [reporting and monitoring]

Award management [process]

Compliance [and reporting]

Grant administration

Grant monitoring, tracking and reporting

Grantee reporting

Grantmaking practices

Grants and awards management

Grant[s] management

Grants management and reporting

Grants program monitoring and evaluation

Grant[s] reporting

In-grant implementation

In-grant management

Management of external research funds

Management of [grants/awards] and reporting

Monitoring

Monitoring and closeout

Monitoring and [outcome] evaluation

Monitoring and reporting

Monitoring compliance

Ongoing assessment

Post-administration requests

Post-award administration

Post-award grant[s] management

Post-award management

Post-award monitoring

Post-award monitoring and reporting

Post-award phase

Post-award processes

Post-award research activities

Project management

Project reporting process

Project tracking management

Reporting [process]

Reporting and research impact assessment

Research assessment of progress criteria based on reported output

Research contracts reporting

Research management

Research reporting

Scientific reporting

Tracking individual projects

### Collating and summarising data

Table 3 includes a summary of the 52 records and the key information extracted from each record. In line with eligibility criteria, all records provided information on post-award management processes. The majority (44 papers; 85%) also referred to the types of stakeholders involved, of which 30 different types were identified and the ‘researcher/principal investigator’ was the most frequently mentioned (20 publications; 38%). Twenty-eight records (54%) also provided information on digital systems used by funders and HEIs to support award management and reporting; in these, we captured 37 systems/initiatives in total.

### Understanding the process

To help simplify the complex process of post-award management and gauge the level of effort that may be involved, the processes, tasks and reporting requirements extracted from records were simplified into a list of ‘items’, and divided into 14 categories (representing the components of the post-award management process (see Table 4)). Items include any processes, considerations or tasks also handled by non-research HEI staff, such as research managers, finance and administrative support staff. ^25^ Based on the number of records citing items in each category, the top five categories were: Award-set up, compliance and close-out, Financial management and financial reporting, Progress reporting, End-of-grant reporting, and Digital tracking of outputs, outcomes, and impacts.

**Table 4.** The components of post-award management and reporting. Extracted information on the post-award processes, tasks, and information requirements (items) is categorised and arranged in a descending order of frequency (number of citing records). Note: Responsibilities for each category or item may vary and are not limited to research staff.

Category	List of processes, tasks and information requirements related to post-award management and reporting (items)	References
Award set up, compliance and close-out	Notice of Award, Grant acceptance and special conditions, Contract negotiation, Account set-up, Reviewing award T&Cs, Subcontract management compliance, Effort reporting (add/remove effort or personnel), Programmatic and financial compliance, Data management compliance, Monitoring and evaluation compliance, Open Access policy compliance, Grant closure and technical closeout, Due diligence, Export control, Animal testing licenses, Institutional concordats, Auditing, Beyond award (Record retention, Property control, Invention control), Institutional Review Board protocol, Invention statement, Ethical clearance, Governance compliance, Subcontract monitoring and compliance, Inactivating projects, Hiring, Travel arrangements, Payment to research participants, technology transfer, statutory returns	^6^^,^^7^^,^^14^^,^^19^^,^^25^^,^^29^^–^^31^^,^^40^^,^^42^^,^^48^^,^^52^^,^^65^^,^^67^^,^^75^^,^^79^^,^^80^^,^^84^^,^^87^^,^^90^^,^^93^
Financial management and financial reporting	Accounting and monitoring award balances (accounts payable and receivable), Payroll, Financial reports, fiscal oversight, Budgeting and budget forecasting, Burn rates, Financial statements (Staff expenses, Payments, processing of Invoices), Financial conflict of interest, fiscal closeout, Final financial accounting/reconciliation, Financial acquittals, Expenditure for contracted services, Subcontract-related invoicing, Record preparation and review, Processing grant personnel (internal and external), purchasing transactions, Just-in-Time requests, Cost sharing/transfers	^7^^,^^79^^,^^80^^,^^85^^,^^87^ ^1^^,^^6^^,^^14^^,^^30^^,^^31^^,^^42^^,^^45^^,^^52^^,^^58^^,^^65^^,^^75^^,^^89^^,^^92^^,^^93^
Progress reporting	Project goal and objectives, Overall Progress, Participants, Milestones and deliverables, Key research outcomes, Achievements and preliminary results, What’s Next, Changes to work plan, Outputs (publications, in-progress publications, non-compliant publications, conference abstracts, books), Authorship, Individual contributions, Presentations, Activities, success stories and testimonials, Barriers to research, Expenditure and justifications, Intellectual Property, Collaborations, Leveraged funds, Patents and products, Courses, meetings and outreach activities, Unexpected events, Personnel changes, Summary of original expected outcomes and planned activities to achieve them, carry over requests, Special reporting requirements	^7^^,^^31^^,^^35^^,^^46^^,^^48^^,^^52^^,^^58^^,^^75^^,^^81^^,^^82^^,^^85^^,^^90^^,^^92^^,^^94^
End-of-grant reporting	Plain English summary, Achievements, Further funding, Engagement activities, Intellectual Property (if relevant), Details on innovation, Collaboration and Interventions/Tools/Services Developed, Impact statement (scientific impact and likely impact from research on the health, economy, community, and policy), Publications linked to grant ID, Summary narrative and how the money was spent, Difficulties that have been encountered, Most challenging/surprising aspects of the project, Advice to others planning a similar project, Strengths and limitations of the project Post-grant plans	^28^^,^^44^^,^^47^^,^^52^^,^^58^^,^^75^^,^^81^^,^^85^^,^^86^^,^^91^
Digital tracking of outputs, outcomes, and impacts	Outputs, Products, Outcomes, and Impact reporting on shared digital and open research platforms (Researchfish, ORCID, Gateway to Research, Pure, Converis, ImpactTracker, REDCap, UKRI Funding Service, AMRC Open Research, Wellcome Open Research), PPI question set, Domestic and foreign collaborations, Publications of any type (consultancy reports, policy briefings, journal papers), Post-award employment (Researchfish)	^14^^,^^18^^,^^19^^,^^34^^,^^37^^,^^38^^,^^40^^,^^58^^,^^95^
Variation requests	Applications for Changes (Research Plan, Principal Investigator, Institution, Commencement Date) Revision of grant strategy or budgets, Extension requests, Changes in Standard of Work or Key Personnel, Changes in T&Cs of award, Notification of approval for changes to research design or objectives, Submitting requests to pre-/post-award offices for project changes/prior approvals	^14^^,^^31^^,^^43^^,^^47^^,^^52^^,^^82^^,^^89^^,^^90^
In-person and informal reporting	Site visits, presentations to programme officers, Site reviews, Informal discussions, presentation of outcomes and results after project completion, Surveys or interviews with principal investigators (e.g., for qualitative impact assessment)	^8^^,^^29^^,^^35^^,^^42^^,^^44^^,^^45^^,^^58^^,^^85^
Performance reporting	Participant enrolment/recruitment (e.g., for clinical trials), Research income, Journal output and Relative Citation Ratio, Measurable outputs (publications, patents granted, higher degrees awarded, conferences or meetings, references in published policy reports/guidelines), preparing data for reporting to the Research Excellence Framework (REF)	^1^^,^^42^^,^^49^^,^^50^^,^^75^^,^^79^^,^^88^^,^^89^
Data management and accessibility	Data management plan (DMP) and teams Completion of institutional data repositories (Jisc, Je-S, Gateway to Research, CASRAI, ISNI, CRIS/IR, Integrated Grants Management System, Integrated financial Management System, Worktribe), Open access policy, Accessibility/discoverability of data, Internal grant approval, preparing accessible reports to support institutional and local decision-making	^1^^,^^14^^,^^39^^,^^43^^,^^51^^,^^67^
Promoting, publishing, and disseminating research	Selecting Open Access-compliant journals, Promoting projects on digital platforms and social media, or using non-digital methods (posters), publication on organisations’ websites/community platforms, Acknowledging source of funding in publications, Dissemination plan (authorship considerations and acknowledgements), employer affiliation, Plain English summaries of articles, ORCID IDs	^19^^,^^44^^,^^48^^,^^86^^,^^92^
Clinical trial transparency	Adding clinical trial to a public trial registry (ISRCTN, clinicaltrials.gov, EU CTR) Reporting to funder the trial registry number Reporting trial outcomes and outputs within a certain timeframe (e.g., within 12 months of study completion)	^28^^,^^81^^,^^89^
Security of research	Reporting foreign influence (Financial conflict of interest, Conflicting IP or authorship, Other support, Time commitment to foreign institutions, Foreign government grants or Personal funding), Data security	^14^^,^^83^
Feedback to funders	Written feedback to funder on award experience for programme improvement	^44^^,^^46^
Assessing researcher performance	Researcher CV data (affiliations, outputs, ORCID ID)	^39^

### Understanding the effort

Based on Table 4, for HEIs the most post-award effort is likely to be around the set up and close-out of research awards. This includes the duties of researchers, RA staff and finance departments, and any other stakeholder involved in supporting the processes associated with these post-award stages. Table 4 presents the processes, tasks and information requirements that could be involved, of which 29 items were related just to Award set up, compliance, and close-out. As an example, some of these are tasks related to providing ‘assurance’ to funders, ^14^^,^^26^ and include due diligence, internal auditing, and reviewing award Terms and Conditions (T&Cs). Others include considerations for funder policies, such as compliance with Open Access of study data, governance, and record retention. Financial management and financial reporting also involve many tasks (17 items; Table 4) required for funder assurance, ^26^ and these include monitoring award balances, managing Payroll, reporting expenditure for contracted services, and producing financial statements.

Reporting is another major focus of post-award effort, based on all the information that may be required for Progress and End-of-Grant reporting ( Table 4). We found 22 items associated with Progress reporting which refer to specific information requirements for report contents (such as the ‘project goals and objectives’, the ‘overall progress’, ‘milestones and deliverables’, and ‘changes to work plan’) and 15 items associated with End-of-grant reporting ( Table 4), which notably include content such as a ‘Plain English summary’, an ‘impact statement’, and ‘post-grant plans’. Some information, such as ‘outputs’, ‘achievements’, and ‘Intellectual Property’, can be requested in both types of report and can also be required for Digital tracking of outputs, outcomes, and impacts ( Table 4) on research platforms such as Researchfish. ^27^ Of note, other categories of reporting that add to the overall effort for researchers include In-person and informal reporting (6 items) and Performance reporting (5 items) ( Table 4). The former may apply to any project and typically involve researchers presenting their progress to programme officers during site visits, or delivering additional information to funders (e.g., on impact activities) via surveys or interviews. Performance reporting on the other hand is more specific to research involving participants (e.g., clinical trials) and involves the reporting of participant data, such as enrolment and recruitment figures, as well as the outputs and outcomes of these studies (e.g., in compliance with funder clinical trial transparency policies). ^28^

### Understanding the purpose

In an effort to better understand the value of post-award management and reporting, and why organisations request information from researchers and HEIs throughout and beyond the award period, relevant information from records was collated into common purpose-related statements (i.e., *“The purpose of post-award management is …”*) ( Table 5). In total, 57 common purpose-related statements were written from collation of data and then thematically grouped into 14 categories of purpose ( Table 5) and arranged in a descending order of frequency (based on the number of citing records). Based on this analysis, the most common reasons for needing a post-award management process were: Research impact assessment (14 records) (e.g., “ *To understand what works in research and leads to impact”*), Compliance (12 records) (e.g., *“To comply with local legislation and the institutional rules that govern research”*), Accountability to sponsors, volunteers and the public (12 records) (e.g., *“To satisfy project sponsors and commissioners”*), Funder programme development and planning (10 records) (e.g., *“For funders to identify gaps where future funding may be needed”*), and Ensuring responsible research conduct (9 records) (e.g., *“To maintain integrity and ethics in research”*). Less common but notable reasons included Securing future funding for researchers (8 records) (e.g., “To maintain funding support and advocate for the need for further research”) and Promoting and protecting the reputations of institutions and researchers (6 records) (e.g., *“To promote the profiles of projects and researchers”*).

**Table 5.** The purposes of post-award management and reporting. Purpose-related statements (n=57) reflecting the common reasons for needing post-award management and reporting in research. Each statement is based on the information collated from records, with the contributing references indicated. Categories of statements relating to a common purpose are shown in a descending order of frequency (based on the number of referencing publications).

	Purpose-related statements reflecting the information collated from publications and reports	References
*The purpose of post-award management is …*		
Research impact assessment	To understand what works in research and leads to impact	^34^^,^^58^
	To provide information on broader changes in society and the translation of research (e.g., impact on health)	^47^^,^^85^^,^^95^
	To connect original research and grantee outputs and products (e.g., Intellectual Property) with new developments in the field	^8^^,^^86^^,^^95^
	For awareness of grantee-reported exchanges with policymakers and public stakeholders (e.g., providing commissioned evidence or serving on advisory committees)	^8^^,^^95^
	To evaluate the impact of research on the number and type (e.g., cross-disciplinary) of collaborations established by investigators as a consequence of the funded research	^77^
	To advocate for the need to continue funding a particular area of research	^34^^,^^58^
	To measure the translational impact of research on local and international communities	^42^
Compliance (regulatory, technical, financial, administrative)	To comply with local legislation and the institutional rules that govern research	^7^^,^^14^^,^^42^
	To ensure day-to-day compliance with funder conditions and requirements during and beyond the award (e.g., data management, audit activities, record retention)	^30^^,^^43^^,^^52^^,^^87^
	To comply with funder monitoring policies (e.g., reporting recruitment milestones for trials)	^88^
	To comply with funders’ fiscal and regulatory policies	^42^
	To ensure proper use of funds for programmatic operations	^45^^,^^65^^,^^75^
Accountability to sponsors, volunteers, and the public	To satisfy project sponsors and commissioners	^34^^,^^48^
	To be transparent and accountable, building trust between funders, research institutions, sponsors and the public	^30^^,^^87^^,^^91^
	To track and account for the spending of public and charitable funds	^14^^,^^38^^,^^49^
	To demonstrate public and patient involvement in research (e.g., as required by Researchfish)	^95^
	To motivate research volunteers (e.g., public members) to continue participating in research by demonstrating the impact of their involvement in research	^48^
	To help governments and public sponsors decide on best allocation of funding resources	^95^
	To demonstrate payback in the form of new knowledge, contributing to research capacity, patient care and political/economic benefits	^49^
Funder programme development and planning	For funders to monitor the successes and failures of their programmes, and identify areas for improvement and learning (e.g., develop training)	^52^^,^^95^
	For funders to obtain information that is critical to their goals and the good of society	^82^
	For funders to identify gaps where future funding may be needed	^40^^,^^58^
	To provide data for annual reporting of funder rates, strategies and impacts, as well as other relevant statistics (e.g., diversity in funding)	^47^^,^^84^
	To use the data gathered to set policies and make the case for more government backing	^19^^,^^84^
	For funders to compare their performance against other organisations	^18^^,^^84^
Ensuring responsible research conduct	To maintain integrity and ethics in research	^14^^,^^52^
	To demonstrate good clinical practice	^48^^,^^52^^,^^88^^,^^89^
	To demonstrate rigour and effectiveness of studies (e.g., statistical power to answer a research question, high standards of work)	^7^
	To protect the welfare of research participants	^89^
	To ensure there is documentation of high-quality research being carried out and standards of good research practice being implemented	^81^
Transparency and dissemination of research outcomes	To report non-academic publications (e.g., consultancy reports, policy briefings and standards papers)	^95^
	To provide information on any changes over the grant period, as well as academic outputs, community engagement, and dissemination activities	^47^^,^^75^^,^^86^
	To report collaborations (locally and oversees)	^95^
	To grow the evidence base and inform the work of other organisations	^48^
	To ensure transparent reporting of studies and outcomes, and to reduce publication bias (e.g., as per clinical trial registration and transparency policies)	^28^^,^^38^
Securing future funding for researchers	To maintain funding support and advocate for the need for further research	^29^^,^^38^^,^^48^^,^^87^^,^^89^^,^^95^
	To help demonstrate success in securing further funding as a reported outcome of the award	^86^
	To build a relationship with the funder through regular communications (e.g., progress reports)	^75^
Monitoring project progress, achievements, and responding to issues	To monitor the progress of projects against targets and milestones, and course-correcting when required (e.g., to help meet enrolment targets)	^52^^,^^75^^,^^88^
	To allow researchers to reflect on how well their research is progressing according to their own plans, and what they have discovered	^75^
	To give researchers the opportunity to ask for help	^75^
	For funders to respond to issues as they arise with quick intervention (e.g., real-time monitoring)	^46^
Promoting and protecting the reputations of institutions and researchers	To promote the profile of projects and researchers	^48^^,^^87^
	To track researchers’ careers (e.g., reporting on post-award employment)	^95^
	To demonstrate good work and responsible use of funds to the sponsor (e.g., through reporting)	^75^
	To demonstrate the performance of HEIs	^42^
	To ensure that there are no issues with compliance that may have legal ramifications or jeopardise an investigator’s, or the institution’s, ability to secure future funding	^6^
Supporting researchers with tasks	For the funder to determine how they can better support the researchers they fund	^52^
	To capture mentoring activities	^47^
	To capture how researchers experienced their awards, including how much time was spent on research or indirect research activities (e.g., preparing grant reports)	^47^^,^^95^
	To improve evaluation and monitoring processes (e.g., by capturing and reducing burden for researchers)	^47^
Reusing information	To reduce duplication and enable data sharing, reproducibility of research, and learning (e.g., by reporting on shared data platforms)	^14^^,^^86^^,^^95^
	For universities to collect information on their research and establish repositories for wider access and reuse of data	^40^
Protecting research from theft	To assess and mitigate potential security risks	^14^
	To prevent researchers from breaching institutional and government laws on ‘foreign influence’ (e.g., disclosing conflicts of interest or of commitment, receiving other support)	^83^
Maintaining focus on innovation	To indicate that innovative research has been conducted (e.g., in end-of-grant reports)	^47^
Improving research management	To gain information on whether/how organisations can improve how they manage research	^49^

### Identifying and addressing the issues

To identify which specific area(s) of post-award management and reporting may be creating perception of unnecessary effort or need for improvement through solutions/interventions (e.g., new methods of information collection, administrative support), relevant information was extracted from publications and summarised in Table 6. We identified 29 records (55%) reporting unnecessary effort or issues in post-award management, including recommendations or solutions for addressing these issues. These findings were summarised for each record and the summaries categorised based on the area of post-award management ( Table 6). The six emerging categories were: Supporting researchers with tasks; Research impact assessment; Data management and accessibility; Reporting and digital tracking of outputs, outcomes, and impacts; Monitoring and evaluation strategies; and Award set up. Out of these, issues were most frequently found in ‘Supporting researchers with tasks’ (7 records), ‘Research impact assessment’ (6 records), and ‘Data management and accessibility’ (5 records). Notably, a generalised category, ‘Bureaucracy in research’, summarised a government-comissioned report covering issues across multiple areas of post-award management in the UK. ^14^

**Table 6.** The areas of post-award management needing improvement. Summary of findings from publications describing areas of post-award management and reporting where processes, or the research culture associated with these, create perception of unnecessary effort or need for improvement (n=28). Proposed solutions to the issues are summarised and include examples of previously implemented solutions or the recommendations of authors. HEI = Higher Education Institution.

**Area of post-award management** *Publication (and funding field)* • Described issues (Perceptions of unnecessary effort or need for improvement) • Proposed solutions (previous specific solutions or recommendations)
**Supporting researchers with tasks** *Corona Villalobos, 2020 (US federal funding of medical research)* ^29^ Described issues: • Many HEIs in the US appoint research administrators (RAs) as part of institutional research management and administration (RMA) support; even so, pressures on compliance and reporting means RAs report significant workload and feel stressed when conducting their duties of managing research grants, updating policies, and supporting researchers through the grant lifecycle. • Some research administrators report not feeling appreciated and respected by their colleagues, who may be unaware of their true contributions to research and as such may hinder them from doing their jobs to support research activities. • Differences in professions, negative stereotyping and diverse backgrounds can challenge relationships between RAs and researchers, creating burden for both. • Failure to communicate and interact beyond work, and a lack of confidence in mutual input, contribute to unhealthy relationships between RAs and researchers, impacting on overall research collaboration and funding success. Proposed solutions: • Researchers and RAs should cultivate healthy and respectful working relationships to effectively manage all aspects of grant administration by tolerating differences in opinions, being aware of individual contributions, and being open to new ideas. • Effective working relationships can be facilitated by nurturing mutual respect and trust, communication, and collaboration. *Decker et al., 2007 (US federal grants)* ^7^ Described issues: • In the United States, over $85 million a year was at one time found to be spent on administrative activities related to research. • Most researchers report that the administrative burden of grant management has increased over the years, and principal investigators now devote almost half their time to indirect research activities. • Submission of grant progress reports is considered the biggest source of administrative burden for researchers, even more so than grant proposal submission. • Knowledge of and compliance with federal agency and local HEI policies, procedures and systems all contribute to the time and effort researchers must commit to grant management instead of research. • The distribution of administrative burden is not equal, with those in public HEIs and medical schools shown to be disproportionately affected. Proposed solutions: • HEIs and sponsoring agencies should streamline and co-ordinate grant management processes to help reduce burden for researchers and administrative staff. • A better balance of administrative workload would allow researchers to commit more time to active research, without compromising on accountability and compliance. • HEI department heads should re-assign some of the administrative work currently placed on researchers and find additional support resources to free up more valuable time for research. *DeMoss et al., 2018 (US federal research grants)* ^6^ Described issues: • There are numerous reports that faculty members feel overwhelmed by the administrative burden and the many policies in research, and are looking for more HEI support with grant-related activities. Proposed solutions: • The University of Michigan’s PART-E program (a research administration onboarding and training program) serves as an example of how simply helping new research staff be aware of and understand the internal administrative resources and post-award functions available to them can help reduce faculty burden and make grant management a more positive process. • An effective onboarding programme would need to go beyond basic orientation and would benefit most from regular in-person sessions, which would encourage the building connections and a more positive overall research culture. *Dresen, 2012 (US federal research grants)* ^30^ Described issues: • HEIs lacking the administrative staff to adequately support post-award functions may have problems with grant-related compliance (e.g., late reporting) • Lack of communication between administrative offices and the HEI community may lead to researchers feeling unsupported and not knowing who to contact in HEIs about compliance issues. • HEIs need to recognise that most researchers and clinicians delivering funded research are not likely to fully understand the complex administrative and regulatory requirements of that research, and may therefore need expert assistance with compliance. • HEIs need to ensure they employ a program administrator or team who understand the issues around compliance and can ensure these are properly addressed for each research award. • There is a need for training of staff and students in grant-related administrative activity that is currently overlooked. Proposed solutions: • An internal ‘research administration’ website – such as the one developed and tested for usability at the University of Wisconsin-Stout Graduate School – could help the HEI community gain the necessary knowledge on compliance and federal/HEI grant regulations. • An easy-to-read and navigate website about a HEI's administrative services should include clear information on where to go and who to contact (e.g., for post-award queries). *Flores-Rivera, 2020 (US federal research)* ^31^ Described issues: • Limited faculty services related to delivering and supporting grant management functions can lead to burden on central offices, researcher dissatisfaction and poor compliance. • Lack of faculty support with pre- and post-award requirements means researchers are having to spend more time than ever away from their research. Proposed solutions: • Holding workshops to assist faculty and researchers with grant requirements (e.g., budgeting) is a scalable approach to achieving a more shared faculty service for support with administrative activity, as demonstrated by the John Hopkins University’s Cornerstone Project • Effectiveness of faculty services and grant compliance activities can be evaluated using general service metrics (e.g., *“percentage of accounts reviewed with a principal investigator in a given month”*) and post-award performance indicators (e.g., *“number of financial reports submitted past the sponsor due date”*) *Hunter et al., 2014 (Environmental and global health)* ^33^ Described issues: • It has been shown that researchers are not always able to understand or comply with some of the funders' monitoring requirements ( such as related to finances and reporting). • Research management is highlighted as one of the most common issues in developing research sectors (such as in Africa), meaning researchers are either unaware of the HEI rules around research management, governance and ethical clearance, or have to do most of the work themselves. • As a result of receiving inadequate support in understanding funder requirements, researchers may agree to comply with contractual requirements that they may then find themselves unable to deliver. • Researchers find themselves unprepared for the time that is needed for certain compliance requirements, such as ethics approvals. • A lack of IT support in HEIs sometimes means that data security rules are inadequately followed, and this results in some grants being withdrawn. Proposed solutions: • Solutions to enhancing research management of externally funded research could involve better guidance for researchers (e.g., on appropriate terms in contracts, finances) and more support from senior investigators. • Better planning of the roles and responsibilities in post-award management (who will do what) can prevent delays to the research. • Where required, researchers should be helped with the recruitment of participants or volunteers (e.g., for community-based projects), particularly with issues such as compensations. *Kasim et al., 2021 (Malaysian research sector)* ^32^ Described issues: • The time researchers spend managing their research can affect their career development, teaching duties and the HEI's performance (outputs and achievements). Proposed solutions: • HEI investment into professional development/upskilling of staff in research management, or hiring dedicated research managers, would remove significant workload from researchers and give them their time back. • HEIs in different regions should share their best practices for managing research to achieve improvement at the local level where it is needed
**Research impact assessment** *Adam et al. 2018 (Health research in Spain)* ^36^ Described issues: • There is inconsistency in how funders and countries approach research impact assessment (RIA). • Despite increased scrutiny into how publicly funded research impacts society, there is a lack of systematised knowledge or accepted standards as to how research impact should be assessed. • There is a need for more transparent measures of non-academic impact and more focus on public participation in research. Proposed solutions: • The Agency for Health Quality and Assessment (Catalonia) propose a ten-point guideline for effective RIA based on expert evidence and opinions at the International School of Research Impact (ISRI), which incorporates ten ‘core values’ of assessing impact: context, purpose, stakeholder needs, stakeholder engagement, conceptual frameworks, methods and data sources, indicators and metrics, ethics and conflicts of interest, communication, and community of practice. • ISRI and other funders who may adopt the guideline should ensure they address any gaps in geographical, disciplinary or stakeholder representation that may limit its effectiveness, and continue to evaluate and improve their RIA practices. • Funders should be explicit at the reporting stage about how they use the impact information provided by researchers for RIA, and notably whether it affects their future chances of funding success. • The UK’s national advisory group, INVOLVE, have addressed the need for more public engagement in research by requiring that all research reports include a plain English summary. *Adam et al. 2012 (Clinical and health services research in Spain)* ^35^ Described issues: • Local and regional knowledge gaps have been identified and need addressing in the evaluation of clinical and health services research. • There is a need for a more complete research evaluation cycle, where funders assess how the research they support impacts on the generation of knowledge, identification of research topics, priority setting, and decision-making. Proposed solutions: • According to a qualitative study by the Agency for Health Quality and Assessment (Catalonia), funders should evaluate the complete research cycle (from identification of knowledge needs to reporting the impacts of individual studies) and engage researchers in identifying opportunities where the whole process can be improved. • Funders should evaluate how the research they fund impacts on advancing knowledge and decision-making. • Funders should put more effort into research on research and knowledge transfer, using a variety of quantitative and qualitative methodologies and indicators to inform RIA. • Funders should harness local evaluation agencies to identify local or regional areas of need, assess the impact of current research on these areas, and refine their research agenda. • the Canadian Academy of Health Sciences Return of Investment (ROI) model suggests that funders assessing impact using quantitative indicators should factor in the interrelationship between research outputs and implementations of findings. *Allen 2016 (UK research)* ^37^ Described issues: • The enormous investments of UK’s research funders into tracked online platforms aim to enhance how we capture the impact of research but may be inadvertently impeding its productivity. • Duplication of effort in reporting research impact on different digital platforms costs researchers and HEIs significant time and effort that is taken away from progress and opportunities in science. • The mass of impact-related data stored across a range of platforms may create complexity for those who wish to measure it (i.e., external organisations, evaluators, and policymakers). Proposed solutions: • Organisations need to think about how to increase the actual impact of the research as well as how to improve how it is reported and assessed. • Organisations need to better recognise the value of sharing and re-using research data. • The research sector should promote connectivity and data sharing across the various reporting platforms (e.g., through ORCID) to reduce burden for researchers and increase efficiency for evaluations and impact assessments. • Funders and HEIs should promote learning through research on research. • HEIs and organisations should agree on common systems and taxonomies for describing and curating impact. • Open access mandates currently applied to publications represent a solution that should be applied to the output and impact data of individual research awards. *Buck, 2014 (UK and other research sectors)* ^38^ Described issues: • Increasing economic pressures and a changing research sector has led to more demand on funders to justify their public spending on research and demonstrate value for money. • Researchers face increasing administrative burden in having to report the same information to multiple organisations and stakeholders (including their funders), which obstructs them from pioneering lab work and disseminating their outputs to a wider audience. Proposed solutions: • There is opportunity for better cross-collaboration and data sharing between funding organisations and HEIs across the research ecosystem. • Advances in IT should be harnessed to replace systems that are no longer effective, potentially inhibit collaboration, or create unnecessary burden for users. • Systems should standardise use of virtual technology where possible, and work towards automatic information acquisition from published output and products. • Horizon 2020 exemplifies a collaborative research and innovation funding program within the EU that led to the creation of Researchfish – an online platform for tracking research outputs, assessing impacts, and connecting openly accessible research information and outputs with specific grants and researchers. • For policymakers, Researchfish data informs research strategy and policy planning; for researchers and administrators, the platform reduces the need for duplicative information requests. • Impact in Researchfish is tracked through imported citations (e.g., from Europe Pubmed Central) and relies on the information given by the researchers themselves. *Collado et al., 2017 (US philanthropic health research)* ^8^ Described issues: • Relying solely on web-based tools to track impact may create a rigid and inaccurate picture for funders and policymakers, in which important but informal examples of impact (e.g., conversations between colleagues, networking) are not captured in the absence of direct and regular communication with researchers. • Researchers report a lag between the end of a research study and the publication of findings, which may not coincide with the window for relevant policy discussions and assessments of impact. • Peer-reviewed publications require follow-up to track impact many years after a study has been completed, adding extra burden for research managers and evaluators. Proposed solutions: • Funders could stratify the impact-related information they collect from researchers to include different types (quantitative and qualitative) of self-corroborating information (e.g., asking researchers to report grant-related products and correlate these with visibility metrics) • Use of multiple web-based tools (e.g., Google News, media-monitoring software), to complement data captured from reporting platforms could enable funders to collect additional useful indicators of research impact (e.g., mentions in social media, citations in policy documents and alt-metrics) • Regularly contacting researchers for a narrative account on their dissemination, engagement and impact activities can give funders more accurate updates on impact than if they relied on annual submission of impact reports (e.g., to Researchfish) alone. Consideration is however needed for the time and effort this regular contact would cost to researchers and evaluators, and that the given information would need to be confirmed by ‘research users’ (i.e., policymakers and other end users) • The Metrics Menu Tool was developed and piloted by the AcademyHealth (USA) as a solution for tracking web-based quantitative and qualitative research impact data in real time. Grant holders were able to provide examples of impact as they occurred, and this led to collection of an enhanced set of detailed impact-related information (albeit requiring additional administrative resource from the funder) *Hinrichs, Montague and Grant, 2015 (UK research grants and contracts)* ^34^ Described issues: • There is a need to invest more effort into maximising the value of Researchfish impact data for the research community; in particular, developing data analysis and sharing, analytical capability and capacity, data integrity and connectivity with the research ecosystem • Unequal funder capacity and capability for analysing data from Researchfish represents a key challenge; smaller funders may not have the resources (staff and software) to develop in-house evaluation processes and systems for producing analytical reports (e.g., on impact data) • There is inconsistency in the completeness, quality and accuracy of data entered into Researchfish, which requires validation so as not to undermine the value of analyses (e.g., impact assessments) • There is room for improving the interoperability of Researchfish with other data systems to avoid the burden of double data entry. However, this cannot be achieved without agreement on data standards and appropriate data validation Proposed solutions: • A solution to funder capacity and capability for data analysis may be to provide training and administrators to those who need it, to use a third party, or set up a ‘consortium’ for analysing data across funders • The importance of entering outputs accurately into Researchfish needs to be communicated to researchers and can be helped by funders being more transparent about how the data they request is being used • Efforts to connect Researchfish with other information systems (e.g., ORCID) and facilitate the validation of data need to continue. Steps have already been made to share information with publication datasets, such as PubMed for instance.
**Data management and accessibility** Burland & Grout, 2016 (UK research) ^39^ Described issues: • In a research sector with a highly varied data management infrastructure, a lack of interoperability and standardisation between systems, HEIs and organisations can create burden for users. • There is growing demand on researchers and HEIs to report on the performance and compliance of grants, and multiple requests for the same information from different organisations means that researchers must manually re-enter it into different data systems. Proposed solutions: • Enabling system-to-system communication between platforms would increase the efficiency and accuracy of reporting, ensuring it is compliant with funder policies and provides HEIs and research managers with consistent impact metrics. • For HEIs, the increase in reporting requirements could be managed more easily by sharing information and promoting consistent use of universal key standards and unique identifiers when reporting outputs. • Specialised research information systems (e.g., Jisc) offer interoperable solutions to better information sharing that are tailored to the UK’s academic sector. • Jisc software enables transparent reporting, communication, and measurement of research data, and currently leads the sector in promoting standards, universal identifiers, and interoperability between data management systems. • Standards in HEI research administration and data management, including use of unique identifiers (e.g., ORCID, ISNI, DOIs), is promoted by the Consortia for Advancing Standards in Research Administration Information (CASRAI) through collaborative projects with universities. • Other systems promoted for HEI use include Current Research Information Systems (CRIS/IR), Je-S, Gateway to Research, open-source solutions for specialist/small HEIs, simple repositories, databases or spreadsheets, CASRAI templates, reusable pick-lists (CRediT, academic career levels) and glossaries for open access research data management. • Funders can also contribute to interoperability and re-use of research information in joint ventures with HEIs and initiatives, as exemplified by the collaboration of Research Councils UK (now UK Research and Innovation) and Jisc on the Overview of Systems Interoperability Project (OSIP) *Clements et al., 2017 (UK academic research)* ^41^ Described issues: • Lack of communication and interoperability between HEI and funder systems (e.g., CRIS/IR and ResearchFish) can lead to errors in information flow and requires duplicative input of information from researchers. • Research information submitted to funders must comply with funder policies and cannot simply be imported from HEI systems (which may cause import errors). • Improvement is needed in the bi-directional flow of information between HEI and funder data management and reporting systems. Proposed solutions: • Automatic data importing would remove the burden of manually entering publication data from researchers. • HEIs should work with funders to transfer grants-linked publications data from internal information management systems to funder-supported platforms. • Automatic transfer of grants-linked publication records from CRIS/IR to Researchfish has been proven to be possible, where almost every record passed validity checks. In the six-institution collaborative pilot, automatic transfer of data prevented researchers from having to manually link around 2,500 publications to grant IDs (reportedly saving them time). *Davidson et al., 2014 (UK research)* ^40^ Described issues: • Universities collect and retain the research data they produce, but this data is often less well curated and can be very difficult to find externally, limiting its value and potential for reuse. • Many disciplines currently lack access to an appropriate subject-specific data centre and the funding support from research organisations to properly curate and encourage reuse of research information. Proposed solutions: • Data management plans and data curation centres can help HEIs better manage their data and researchers track their research outputs, as well as understand the ethical and legal requirements for dissemination and reuse. • The UK pilot research data registry and discovery service (RDRDS) is an example of an initiative that can help ensure that research data produced by HEIs can be found, understood, and effectively reused. • Funders should explore the value of data registries and discovery services, such as the RDRDS, for their own research data tracking, assessment and sharing needs (e.g., for reviewing the impact of funding and identifying trends and gaps). *Lehman, 2016 (US sponsored research)* ^42^ Described issues: • A frequent problem in the management of sponsored research is the use of homegrown or proprietary information systems that are separate, non-integrated, or inadequate. • Duplication stems from issues with system interoperability that span the lifecycle of research awards and may represent a key source of burden for researchers. Proposed solutions: • As identified by a Delphi consultation, a critical factor for research management success is having a common ‘enterprise-level’ information system that can integrate and work across multiple existing HEI departments and systems (e.g., finance and Intellectual Property). However, achieving this would require additional funding and resource for system maintenance and regular software updates. *Sundjaja, 2019 (Indonesian research grants)* ^43^ Described issues: • It is suggested that advances in IT are currently insufficiently harnessed to tackle administrative burden and inefficiency in research sectors, which leads to slow progress in improving HEI information repositories and grant management systems. • Absence of internal grant management software and reliance on manual input (e.g., in Excel) makes collating and process of data, as well as monitoring of grant status, complicated and time-consuming for HEIs. Proposed solutions: • Consulting researchers (as well as research coordinators, grant administrators and reviewers) on areas where managing grant information can be improved using software can help HEIs adopt new, or improve existing, systems. • What researchers, administrators and reviewers reportedly require from internal grants management systems is usability (e.g., easy registration of proposals, assessments or document and report checks) and an overall efficient flow of information throughout the grant cycle.
**Reporting and digital tracking of outputs, outcomes, and impacts** *Abdullahi et al. 2021 (Small grants in Kenyan health research)* ^44^ Described issues: • Funders often require grant holders to carry out and report on stakeholder engagement but may not always follow through with adequate support with encountered issues (e.g., political, administrative) when trying to engage with certain types of stakeholders. • Funders could easily learn where they need to better support their awardees by asking for feedback at the reporting stage. Proposed solutions • The RHD Action Small Grants Programme learned of and was able to respond to grantees’ issues with engaging government stakeholders by requesting feedback on the funding programme and recommendations for improvement as part of the reporting process. *Knowles et al., 2020 (UK medical research)* ^28^ Described issues: • There is concern of inadequate researcher compliance with funder policies regarding transparency of reporting clinical research, and that some researchers may be selectively reporting positive findings. • Funders may need better ways of encouraging researchers to report trial findings transparently and reliably within a certain time window. • Having to collect and curate a reliable set of trial data from multiple sources (grant applications, grant management and reporting systems) poses a challenge for funders. • Funders have yet to universally adopt a trial registration policy to facilitate transparency of reporting and collection of trial data. Proposed solutions: • Trial registries are now supported by many funders and significantly reduce bias in outcome reporting and publication; as such, monitoring compliance with trial registration should be the focus of future funder policy initiatives. • More funders should encourage researchers to register their trials on public platforms (e.g., ISRCTN, EUCTR, ClinicalTrials.gov ) and report trial registration numbers, along with updated details of outputs and protocols, to increase transparency of reporting. • Contacting researchers to remind them of the reporting window is shown to be effective in prompting them to report or publish findings. *Muller, 2009 (US federal research)* ^45^ Described issues: • Overreliance on technology in grant management can lead to duplication of effort for researchers when reporting research data. • Organisations are asking for more information and documentation to be reported, creating extra administrative costs, taking time away from program delivery and creating burden for researchers. • There is a perception that organisations could be clearer and more upfront with researchers about how the information they request at the reporting stage is used and who uses it. • Researchers report a lack of guidance and communication from funders regarding reporting requirements, and the timing of information requests is said to be problematic. • Effort in reporting is affected by factors such as the researcher’s workload and experience, the funder’s specific requirements, the administrative capacity in HEIs, and availability of staff training. Proposed solutions: • Based on the opinions of funding organisations and grantees, the level of administrative burden in compliance and reporting could be better monitored by funders as part of their evaluations by capturing ‘metrics’ such as “ *number of reporting requirements*” and “ *length of time in providing requested information*” along with the other information collected in reports. • Funders should streamline their grant management processes to reduce burden, ideally achieving a balance between ensuring accountability and considering researchers’ interests. • Funders should consider what information they require as minimum, and concentrate their monitoring activities on higher-risk grants • By adopting a ‘triage’ (i.e., risk-proportionate) approach to grant management, funders can free up capacity to provide extra support to those who need it most, such as new investigators. *Sajdyk et al., 2014 (US Federal clinical and translational research)* ^46^ Described issues: • Reporting and collecting research information can take hours or days and creates administrative burden for researchers and programme managers. • The time it takes to complete reports means stakeholders may not have access to the most current research information. Proposed solutions: • HEIs could reduce the time it takes to report to just minutes by using integrated software systems of data collection. This was exemplified by the Indiana Clinical Translational Science Institute’s implementation of the REDCap integrated grant management system. • Collection of progress reports in real time would allow programme managers to intervene quickly should issues arise.
**Monitoring and evaluation strategies** *Croxson, Hanney and Buxton, 2001 (UK health-related R&D)* ^49^ Described issues: • Economic pressure on the health research system means that putting funding into research instead of directly into healthcare needs to be well-justified and demonstrate return in terms of performance. • Routine methods of managing and measuring performance (e.g., the use of standardised indicators) may not be the best way of monitoring relevant and valuable outcomes from research due to the difficulty of applying uniform indicators across the breadth of health areas and research activities. • Performance measurements tend to focus on measurable outputs (e.g., publications), and not outcomes that are more variable but indicative of improvements in health. • In measuring research outcomes, the time lag between the publication and impact of outcomes is not factored in and can be up to 20 years. Proposed solutions: • The UK Economic and social Research Council proposed a performance monitoring system (REGARD) for health-related research that is not based on uniform targets and indicators, but instead aims to meet five key criteria: that is relevant to funder objectives; it is decision-relevant; it encourages ‘truthful compliance’; it minimises unintended consequences; and it has acceptable net costs. • Utilising the above or a similar system would require the following methods of collecting research data: questionnaires to researchers and user surveys; harnessing bibliometric databases; analysing policy documents; expert review; case studies and economic evaluations. • As a caveat, some information can be collected regularly on all research funded activity, while some would only be collected on a sample of activities. Moreover, posing questions and making accurate assessments on certain activities (eg., policy and health impact) may be difficult but is feasible with the use of questionnaires, based on previous work. *Guthrie et al, 2019 (Australian health research)* ^47^ Described issues: • Funders need to reduce burden and foster fairness, innovation, and creativity in their evaluation and grant management methods. • Current availability and accessibility of data that is used for evaluations is insufficient and should be improved. • Previous funder efforts to evaluate researcher burden, innovation and fairness in grant funding processes have been limited and focus on the pre-award phase. Proposed solutions: • Evaluation measures could better reflect the effort of researchers if they captured the relevant data. Metrics of administrative burden in grant management could include “ *time spent by researchers and HEIs preparing grantee variation applications, financial acquittals and end of grant reports*” and “ *hours of internal administration time and total administrative costs*”. • Funders should ask researchers about their experience with grant programs and where processes (e.g., usability of data systems) related to grant management can be improved. • RAND Australia have shown that metrics could be used to improve evaluation of grant programs at the National Health and Medical Research Council (NHMRC), and have led the design of a new framework to increase the efficiency and accessibility of grant management systems, support innovation and researchers, and allow more time for high-quality research. • The NHMRC has shown that changing grant management systems (in their case, from RGMS to Sapphire) can help improve usability and efficiency for users, saving valuable researcher time through easy features such as the ‘autosave’ function and live character counting. The next step for Sapphire is to connect its research data with external sources, collect more free-text responses from researchers, and provide more flexible online formats. *Bates & Jones, 2012 (UK public health)* ^48^ Described issues: • Community projects often rely on volunteers for delivery and lack the budgeting, staff, or experience for implementing formal monitoring and evaluation processes. • There is not enough awareness among community-focussed researchers of the importance of evaluation and measuring research performance and outcomes. Proposed solutions: • Commissioning and funding organisations should help the community project researchers they sponsor carry out evaluations. • Funders should clarify for those involved in community projects the reasons why they should be monitored and evaluated (e.g., to satisfy sponsors, inform future work), and outline the key components of a successful evaluation process (e.g., through a guide). Solutions should also be applied to any barriers or problems identified by the research groups. • Community groups should plan for monitoring and evaluation as soon as possible (e.g., deciding early if external evaluators are needed), and consider the different types of evaluations, what they should be evaluating (what outcomes or indicators), and when data should be collected. • The findings of monitoring and evaluation should be widely shared with other community groups and organisations, making use of online resources and social media. *Nature, 2021 (UK and global research)* ^50^ Described issues: • Current evaluation systems (informed by reported research information) use criteria that can create bias and insufficiently reflect unforeseen circumstances (such as Covid-19) that may impede research progress and disproportionately disadvantage some groups (e.g., early-career researchers and female investigators). • Current funder evaluations may be capturing a limited set of performance metrics (e.g., focusing on journal output and research income) and should include other activities, such as mentorship, team building, and opportunities for underrepresented groups). Proposed solutions: • Funders must better recognise their responsibility for having effective research evaluation policies as a step to designing better systems of how research and researchers are valued, measured, and assessed. • Funders could learn from the impact of the Covid-19 pandemic on research performance to inform development of more equitable and balanced evaluation systems.
**Award set up** *Riechhardt, 1998 (US federal research)* ^51^ Described issues: • Long delays in post-award disbursements of funds can create burden for researchers and delay the research. • Delays to research are contributed to by internal bureaucracy and a duplicative HEI approvals process (e.g., including too many people in the approval queue). • Researchers also have to deal with late payments, long grant renewal periods, and an unpredictable grants process. Proposed solutions: • Solutions to ‘post-award lag’ and inefficiency that funders and HEIs could implement include switching to electronic grant tracking and electronic accounting systems, standardising forms and deadlines for funding calls, simplifying the approvals process, and employing more staff to deal with backlog. • Funders should shorten grant renewal periods and remove the requirement for researchers to submit new proposals if only a small portion of their funding requirements (e.g., up to 20%) have changed. • An example of previous reform to grants management processes to reduce post-award lag and administrative burden for grantees is the Space Science Programme at NASA. Programme leaders responded to the burdens identified by researchers by sending out letters, holding open meetings and assembling advisory groups with grant managers at other federal agencies.
**Bureaucracy in research** *Tickell, 2022 (UK research)* ^14^ Described issues: • There is a general perception across the UK's research sector that bureaucracy and burden have increased over time. • Complex and duplicative reporting requirements, slow internal approval processes and non-interoperable data systems have been identified as major issues. • Scrutiny of assurance processes has steadily increased, and new organisational requirements that have served their purpose are rarely removed. • The biggest contributors to administrative burden are HEI bureaucracy and internal delays (e.g., due to unnecessary hierarchy in operational/approval processes and lack of dedicated departments). Moreover, complex assurance and reporting processes perpetuate a culture of ‘risk aversion’ among organisations. • Particularly post-Covid, there is a need to reduce pressure on researchers and increase the efficiency of direct and indirect research activities. Proposed solutions: • Reform in the sector should focus on harmonisation (e.g., standardised data templates, interoperability of data and alternative CV’s), simplification, proportionality, flexibility (e.g., repurposing funding), transparency (e.g., clarifying the need for information requests), fairness (e.g., removing barriers to entry in funding), and sustainability. • Shorter applications, shorter review windows and funding decisions, and wider use of preprint platforms for quicker dissemination of outputs are recommended to improve efficiency. • HEIs and funders should both work to standardise some of the internal assurance, due diligence and reporting processes, and promote information sharing. • Periodic assessment of overall HEI research performance could replace project-level scrutiny and make assurance more efficient. • Funders should employ a principle of ‘ask once’ to reduce duplication in assurance and monitoring processes. • Funders should give HEIs and researchers more time post-award for project set-up (e.g., finances, recruitment) and respond quicker to project change requests and no-extensions. • Funders should explain the purpose of different information requests to researchers. • Funding councils and charities should coordinate their processes and support HEIs with award management and assurance. processes to improve efficiency and reduce burden (e.g., by exploring self-certification). • Funders such as NIHR, UKRI and Wellcome should consider replacing older digital platforms with a unified system that is simple to use and supports organisational diversity while keeping pace with the evolving sector. • HEIs should align their data management practices by transferring to the Jisc system. • Organisations and regulators should consult the research sector before implementing new requirements to agree on the best approaches.

The issues identified not only concern post-award processes, but the culture around these as well. There is a general perception of unnecessary effort and little support in post-award management, and that numerous areas of the process could potentially benefit from tailored solutions (e.g., new systems, resources or streamlined approaches) to reduce burden or add value to research ( Table 6).

### Supporting researchers with tasks

The need to better support researchers with post-award tasks was frequently cited. ^6^^,^^7^^,^^29^^–^^33^ This stems from reports of significant workloads, a stressful environment in HEIs and issues in communications and working relationships. Solutions to these depend on individual HEIs and could range from hiring research administrators (RAs) or improving existing RA-researcher working relationships, ensuring these are collaborative and respectful of the different roles in managing and effectively delivering reserach. For researchers, a better balance in administrative workload would in turn free up more time for research – particularly in overwhelmed settings like public HEIs and medical schools. This would also ensure that funder investments primarily cover the research and not grant-related administrative activity, which would translate into timely research and quicker impact on end-users. Investing in RA support should also help with compliance issues (e.g., late reporting and consequent sanctions), which can occur when HEIs are overburdened with grant management tasks or where researchers are not fully aware of the funder requirements. Organising training or onboarding programmes for faculty, or employing research managers with knowledge in compliance, are therefore potentially scalable solutions that could widely apply to different research settings and disciplines. The efficacy of these solutions, and HEI grant management functions in general, could also be monitored and measured with metrics – to identify where more support with grant management and other management issues in research (e.g., ethics, recruitment) are needed.

### Research impact assessment

The need to improve methods and culture around research impact assessment (RIA) emerged as another key issue in post-award management. ^8^^,^^34^^–^^38^ Despite increased focus on RIA in research, the criteria applied by organisations to measure impact and the methods of gathering award data for RIA may need improvement. Funders are urged to develop better RIA guidelines and address gaps in their frameworks, such as by building on standard output-based metrics (e.g., number of publications) with qualitative examples of impact activities. There is also a need for transparency as to how impact reports may affect the researchers, and to ensure that research assessments include some participation of research end-users. Focus on non-academic impact, and to evaluate the complete research lifecycle, are also encouraged for demonstrating the impact of research on priority setting and funding decisions. At the same time, organisations are urged not to over-focus on impact reporting to allow more time for the research and improve the impact of research itself. To that end, recognising the value of sharing and re-using research data were also suggested, which would help towards developing common systems, taxonomies and methods of data curation for RIA. Such efforts should be collaborative and maximise the value of existing digital platforms (e.g., Researchfish) to ensure completeness and quality of the impact data reported in these. Where needed, funders should also ensure they have the expertise and capacity to effective use these platforms to inform their evaluations and RIA activities.

### Data management and accessibility

Commonly reported were issues with the standards and sharing of research data, as well as its curation and management. ^39^^–^^43^ With information requirements increasing, but organisations using different and non-interoperable digital systems to store and access the data, researchers find themselves spending more time on reporting due to having to manually input information into open repositories, tracked platforms, and funder and HEI grant management systems. In turn, issues in the transfer of information between systems can lead to errors and duplication in data, creating further burden to users. As such, there is a need for more efficient bi-directional flow of information between HEI systems and those accessed by others (e.g., research sponsors). These include widespread adoption of data standards and interoperable systems by HEIs, and automated data transfer and grant-linking of outputs. HEIs and funders could also consult data centres and switch to enterprise-wide information and grant management systems, so that all staff are trained and have the access for efficient management of award data.

### Reporting and digital tracking of outputs, outcomes, and impacts

Funders asks that researchers report on awards in a variety of methods/formats, from submission of written progress reports to updating online forms (e.g., Researchish) with the outputs and impacts of studies. However, where there is overreliance on technology this reportedly leads to an increase in reporting requirements, with the extra administrative effort created leading to delays in research and completion of research reports. ^28^^,^^44^^–^^46^ The need for the information requested is also not always clear, nor are the reasons why funders choose certain reporting strategies over others. Funders could consider what they require as minimum and strive for ‘risk-proportionate’ monitoring, focusing post-award support on those who need it the most (e.g., early career researchers) and being transparent with researchers about how the information they provide is being used. There is also opportunity to ask researchers for feedback at the time of reporting – for instance, to monitor administrative effort in post-award management or learn where researchers require more support (e.g., stakeholder engagement). Resorting to real-time reporting is another solution that could potentially take the pressure off researchers, lead to better information (e.g., examples of impacts) and enable faster funder responses to any issues with research.

### Monitoring and evaluation strategies

Increasing effort in research is put into ensuring that funders’ investments are justified and demonstrate sufficient return in terms of performance and impact. However, how funders themselves evaluate and report these may not result in accurate indicators of value and outcomes from research, and will not work equally well across all disciplines and research activities. ^47^^–^^50^ For instance, there is a perceived focus on output-based indicators, such as number of publications, in evaluating performance; however, these will not necessarily correlate with the true benefits of research to health and society. Other factors that are frequently overlooked but may impact the fairness of evaluations include the time lag between outputs and impact (which can be up to 20 years) and administrative burden in the post-award phase. Solutions in the form of improved evaluation strategies have been suggested; for instance, using qualitative indicators of performance or capturing metrics of effort in research and grant management (e.g., “hours of internal administration time”). In developing more equitable and balanced evaluation criteria, funders should also ensure that some groups of researchers (e.g., female investigators) are not disadvantaged, and that performance or research assessments account for unforeseen setbacks to research delivery (e.g., as in the case of Covid-19).

### Award set-up

One record described historic issues associated with the set-up of NASA-funded awards; these included significant ‘post-award lag’ in the form of late funding disbursements, a long bureaucratic approvals process, and delay to research. ^51^ These issues, affecting the Space Science Programme, led to grantees labelling the entire grants process ‘unpredictable’ and further caused them post-award burden in the form of a protracted process of grant renewals. Ultimately, NASA achieved reform to the programme and solutions included simplifying the HEI processes required for approvals and set-up, digitising grant tracking and finance, and standardising forms for employees. Shorter grant renewal periods were also given to those requesting minimal changes to funding requirements, and feedback was sought from researchers to inform improvement discussions with other federal agencies.

### Bureaucracy in research

Tickell’s large-scale review of UK’s research bureaucracy identified numerous issues in post-award management. ^14^ These were relevant to the processes of both government funders and HEIs, and included unnecessarily complex and duplicative assurance and reporting requirements, as well as inefficiency in the use of data systems, and administrative pressure on research activities. The biggest contributor of burden in HEIs was in most cases a long and bureaucratic approvals process, while funders were found to place unnecessary scrutiny on assurance and therefore added reporting requirements. Recommendations to the whole sector included simplifying and harmonising data systems and focusing on proportionality and flexibility in grant processes and research assessment. Funders were especially encouraged to streamline their assurance requirements and the HEI system to share their research and data management practices.

### Funder websites

Consulting the websites of funders ( Table 2) revealed that all websites included information on post-award management processes, although to varying degrees of detail. Overall, we noted considerable variation in the funders’ approaches to monitoring and reporting: differences included the terminology used to describe post-award processes (e.g., ‘scientific reporting’, ‘project management’), specific reporting requirements (e.g., progress reports) and the frequency of reporting (e.g., annual, quarterly), the type of information requested (e.g, impacts) and where it must be reported (e.g., end-of-grant reports vs platforms), and the digital platforms used to support applications and award management. There were also differences in the level of detail relating to guidance and supporting information for researchers and HEIs (such as policies, justification for requirements and explanations of why information is asked for, how it is used, and who uses it). The main points of variation in practices are outlined in more detail below.

### Reporting

• For most funders, specific reporting requirements and the frequency of reporting depend on the grant scheme or funding programme, meaning that the information asked of researchers can vary. Funders may also vary their requirements on a case-by-case basis or use a ‘risk-proportionate’ approach – for instance, as done by the National Institute for Health and Care Research. ^52^ Some funders, however, may use the same approach to monitor all their awards and we found this is to be the case for the Canadian Institutes of Health Research, Alzheimer’s Research UK, the National Research Foundation, and the National Institutes of Health.
• Most funders require submission of periodic progress reports as part of routine project monitoring. As an exception, the Canadian Institutes of Health Research require submission of a single electronic grant report at the end of a study, while the Medical Research Council generally asks that researchers submit study updates annually via Researchfish (although they may ask for updates on progress using other methods).
• In addition to completing progress reports and publishing in journals, some National Institute for Health and Care Research (NIHR) funded researchers must also publish their full study outcomes and outputs on NIHR platforms, namely the NIHR Journals Library (NJL) or the NIHR Open Research platform (depending on programme). Recently, the NJL has transitioned from publishing full study reports to a flexible ‘threaded publication’ approach, ^20^ where for some studies findings can be published as smaller reports and followed by a synopsis of all study outcomes after study completion. Notably, health and social care studies funded by NIHR must also report to the Health Research Authority, and this is in line with the ‘Make it Public’ transparency and openness strategy that the UK now applies to all publicly funded health research.
• In addition to end-of-grant reports, the National Health and Medical Research Council in Australia requires that fellowship award recipients specifically also submit a single-page summary of the research, and all awardees are also required to annually update their electronic CVs to reflect latest grant outputs as part of routine ‘performance reporting’.
• Most of the funders (7 out of 11) require funded clinical trials to be prospectively registered on at least one public trial registry platform (e.g., ISRCTN ^53^) and for trial results to be transparently shared within a feasible time frame of study completion. For the National Institute for Health and Care Research, clinical trial investigators are also required to submit ‘performance reports’ to England’s Clinical Research Network, so that data such as recruitment can be reviewed against national benchmarks and used for publishing annual performance statistics. Trial and intervention study applicants to the Health Research Council New Zealand, on the other hand, are required to plan their own monitoring as part of application and in advance of funding decisions, as studies of this type must undergo periodic review of safety/efficacy and requests for appropriate panels must be made in advance of the project delivery phase.

### Digital systems

• Apart from the Canadian Institutes of Health Research and University Grants Committee Hong Kong, most funders use their own in-house digital systems to manage applications and funded awards, as well as for receiving and managing research reports.
• Three funders – National Institute for Health and Care Research, the National Institutes of Health, and the European Research Council – each currently use more than one in-house system for managing studies; however, it must be noted that use of systems can be subject to change, for instance, as funders undergo restructuring or as part of funding programme improvement.
• The National Institute for Health and Care Research and the Medical Research Council both require that researchers register their studies, and annually report their outputs, outcomes, and impacts, via the shared platform Researchfish. For both funders, this is a compulsory reporting requirement for all funded awards that is used for monitoring and research impact assessment in addition to other reports.
• In Singapore, all publicly funded research is managed under a single digital system, the Integrated Grant Management System. This system is accessible to all funders, HEIs and researchers and is used for submission, management and tracking of all funding applications and research reports.

### Monitoring and evaluation policies

• With the exception of one funder – the University Grants Committee Hong Kong (for whom this information was not found) – all the funders have dedicated web pages for monitoring and reporting requirements. These include guidance and relevant policies, and in some cases detail on what information is asked for (e.g., downloadable report templates). However, the level of detail and notably the focus of policies related to monitoring vary. For instance, the National Institutes of Health and the Canadian Institutes of Health Research both focus their monitoring policy on clinical trial registration and transparent reporting of outputs, whilst the policy of Alzheimer’s Research UK focuses on research impact assessment, and the National Health and Medical Research Council’s on evaluation strategies and ‘innovation’ of grant management practices. Most funders however make sure to update their monitoring policies regularly, although whether this is the case for the National Research Foundation and the European Research Council was unclear from their websites.
• Most of the funders share downloadable report templates on websites, or provide a summary of the type of information they request from researchers. Alzheimer’s Research UK and Health Research Council New Zealand, on the other hand, do not seem to share their report contents on websites, which suggests they may send them directly to grantees (for instance, once the reporting window is open).

### Resources and support

• All funders give some indication on their websites as to who in HEIs may be best placed to fulfil certain compliance and monitoring activities (e.g., a project director).
• All funders provide a list of relevant offices and research managers/administrators who researchers and HEIs can contact for information or assistance with managing their research awards.
• Awardees with the National Institute for Health and Care Research receive monitoring support from dedicated research managers and teams, who are specifically assigned to monitor and risk-assess funded contracts and support their researchers with fulfilling reporting requirements. As an example, support is provided by sending researchers reminders of upcoming deadlines for progress and final reports. ^52^
• The Medical Research Council employ a Translational Research board and Research Funding Policy and Delivery team, both of whom help manage awards and respond to researchers’ enquiries or variation requests.
• The National Institutes of Health provide their grant recipients with a ‘Welcome Wagon’ letter as part of early post-award communications, which includes helpful information and resources to help them set up their research and manage their research awards.
• The National Research Foundation in Singapore have a web page with guidance and training videos on award management specifically for researchers.

## Discussion

Post-award management is basic condition of funding that serves many purposes but varies in the mechanisms and administrative effort involved. This section discusses the findings of the first scoping review on this topic, focussing on their implications in terms of effort in post-award management, the responsibilities involved, and the support that can be provided or remains needed. We also discuss the availability of evidence in this space, limitations, and future directions for research, and offer broad recommendations for both funders and HEIs.

### Implications of findings

Managing funded research involves more than the signing of contracts and completion of progress reports. The landscape of post-award processes and conditions for funding is complex and there is no relationship between organisation or award type and the approach to reporting. Cataloguing and summarising the available evidence however allowed us to better understand processes and gauge where most of the effort may lie. We were also able to highlight areas where effort may be perceived as unnecessary and improvements are needed, focusing on solutions and recommendations that are relevant to funders, HEIs, and researchers.

For HEIs, significant effort is needed for compliance and the post-award set up of studies, which involves setting up the conditions for the award, obtaining necessary approvals and arranging timely funding disbursements, ^54^ as well as ensuring that the correct infrastructure is in place for responsible and compliant management of finances and research data throughout the award. Notably, research has become increasingly digitalised, ^55^ not least because of Covid-19, and HEIs now store vast quantities of research data which they must ensure is accessible, discoverable to others externally and standardised ^39^ for effective sharing and reuse by the research community. However, siloed approaches to managing data – where funders and HEIs all use their own systems – has led to an overwhelming presence of digital platforms, of which 36 were captured in this review alone and most of which lack interoperability, resulting in duplication of effort for users. ^56^ Although numerous collaborative initiatives ^39^^–^^41^ (such as Jisc and Current Research Information Systems-Institutional Repositories (CRIS-IR)) now provide HEIs with solutions for better system interoperability and data sharing with funders, they have yet to be standardised across the research sector ^14^ and there is still room for reducing manual effort in and improving the transfer of grant-linked research data between systems. Moreover, while HEIs are encouraged to engage with specialised data services to improve the accessibility and reuse value of the data they hold, evidence that funders also engage with these services seems to be lacking ^40^ and inconsistency in how funders themselves use technology for tracking research outputs, outcomes, and impacts may explain why research data is still not being efficiently shared across sectors, ^28^ perpetuating unnecessary effort and research waste for users.

Importantly, too many digital sources of data can also affect funders’ abilities to perform research impact assessments (RIA), ^57^^,^^58^ which in today’s ‘accountability climate’, are crucial for demonstrating that research impact is ‘measurable’ (e.g., resulting in new policy or technology) and for the continued support of the funders’ research programmes. ^34^^,^^58^^,^^59^ While the specific reporting requirements of funders may vary, we found they consistently request that researchers anticipate and report on the impacts of the research they fund. Moreover, the reported information must be relevant and updated after study completion, and as such tends to be collected frequently and using multiple methods, including progress reports, end-of-grant reports, impact statements, and online submissions to tracked impact platforms (such as Researchfish). However, research has shown that having a ‘plethora’ of data sources available to funders for RIA does not mean that assessments are always useful to funders, and there remains no consensus on RIA frameworks, or the meaning of ‘impact’. ^35^^,^^36^^,^^58^^,^^60^ This brings into question the end value of impact reporting to stakeholders, and of adding more effort to this activity on both sides of the award. ^58^ For instance, while there is evidence that new frameworks and tools for capturing broader impact data are being developed, ^8^ the ‘value add’ of these versus the costs (in terms of money and effort) should be also considered to avoid placing unnecessary burden on routine funding operations or on the delivery of research activities.

Researchers already report struggling with routine reporting requirements, ^61^ as well as the multiple systems used to track research data and having to manually link study outputs with the identifiers of research awards. ^39^ With respect to the accuracy of the data reported, some argue there is still room for improvement ^34^ and for funders and HEIs this may mean training researchers in ‘impact literacy’ ^62^ or explaining more clearly the type of impact information they should be reporting. The need for certain types of reporting, such as progress reports, and the need to include impact data in these reports is also up for debate, as it is suggested that funders mostly rely on end-of-grant reports or tracked platforms to collect data for retrospective analyses of the overall and long-term impacts of studies. ^58^ Indeed, we found that not all funders require progress reports of researchers, and reducing effort in this area may therefore mean giving researchers more autonomy as to how they update funders on the progress of their awards – for instance, allowing them to report in real time or through more direct communication channels with funders. ^8^

### Responsibilities and support

A lot of the post-award effort discussed in this work is shared between researchers and many other staff within funders and HEIs, who help coordinate and deliver the complicated post-award management process. ^63^^,^^64^ However, we found that specific responsibilities for requirements, and the level of support offered to researchers in HEIs, depends on availability of research management and administrative (RMA) infrastructure (such as availability of RAs) and other factors in institutional set up. ^6^^,^^7^^,^^29^^–^^32^^,^^65^ As such, while certain award tasks, such as negotiation of contracts and hiring, can be delegated to relevant HEI departments (such as Human Resources and Finance) the level of support offered for other activities – such as review and approval of research operations, managing direct information requests and reporting – is not always clear due to differences in HEI facilities, resources, and internal funding. In addition, RMA appears to heavily vary by country, ^65^^–^^67^ and even where it is readily available (such as in dedicated ‘grant offices’ or ‘post-award offices’ ^3^) a ‘systematic problem’ of administrative burden and issues with compliance is still being reported, ^68^ with issues stemming from factors such as overburdened central offices, ^31^ poor leadership, ^42^ inadequate training, ^30^^,^^65^ and ineffective relationships between researchers and administrators. ^29^ The concern therefore is that not having the needed support for post-award tasks may affect the timely delivery of the research and reduce its impact, as well as return on the funders’ investments. As such, adoption or improvement of RMA and grant support functions in HEIs may be necessary, with the onus then on governments and funders to deliver the infrastructure and training required, ^69^ investing in better research support to fund better quality research. ^70^

### Availability of evidence

There is evidence that strategies to improve funding systems now include efforts to optimise grant management processes ^15^^–^^17^^,^^47^^,^^71^^,^^72^; however, as a research area, we believe that the post-award phase may still be in its formative stage. Evidence on post-award practice is limited in scale, robustness and focuses on interventions (e.g., training, alternative mechanisms) compared to topics like grantsmanship and peer review, ^11^^,^^73^^,^^74^ and the literature aimed at researchers is mostly on improving the quality of grant applications, and not on what happens post-award or improving post-award skills (such as writing of progress reports ^75^).

Most literature in the post-award space also focuses on research impact assessment (RIA) and tends to be high-level, exploring RIA frameworks and strategies in isolation of the reporting that funders require (see Refs. 59, 76 for examples). We found only six publications ^8^^,^^34^^–^^38^ that linked RIA back to the funders’ methods of information collection, and which considered the feasibility of funders being able to collect certain types of data (e.g., qualitative impact data) for the purpose of monitoring and RIA activities. These publications were useful as they showed how some solutions for funders (e.g., improving the accuracy of impact data) may affect individual reporting requirements (e.g., the need for telephone interviews with researchers) and the implications on effort for the evaluators and researchers directly involved in reporting. ^8^ However, literature focusing just on how research is monitored by funders is sparser, and a large proportion of information had to be gathered either directly from funder websites or funding reports, or from publications and theses on award management systems. Much of the evidence on perceived unnecessary effort in post-award management was also gained from grey literature (e.g., opinion pieces ^37^), with little observational data available, such as from faculty interviews and surveys with staff (as in Ref. 29).

Ultimately, we believe the reason for the lack of research in monitoring and reporting is simple: that feeding back to funders on research that has already been funded is generally seen as well-justified and less onerous than applying for funding or undergoing grant application review. For instance, we found that no record argued against funders needing to oversee their research investments in general, and the literature instead provided a catalogue of reasons why monitoring research is important to numerous stakeholders within and beyond academia. As such, despite how researchers may experience the effort that goes into managing research and complying with funders’ requirements, we and others believe they are still likely to see this effort as a ‘necessary burden’ in funding, which will seldom deter them from applying for funding or continuing working in research. ^3^

Nevertheless, it is important to raise awareness of any unnecessary effort or issues with practices that have been accepted in the past but are now impacting on efficiency in funding processes or today’s research culture. To that end, the research sector will benefit from this review of previous work, and more exploratory research, ^42^ independent reviews, ^14^ needs assessments ^33^ and systematic comparisons of practices. Notably for funders and HEIs, strategic changes should focus on ‘grant implementation’, ‘in-grant management’, and ‘digital platforms’, ^14^ and the success of any future interventions (whether it is guidance and training or integration of new systems) should be prospectively evaluated (such as in Ref. 77) or followed up to determine the long-term effects as the research sector evolves and new burdens arise. Continuing to capture ‘effort’ and the experiences and perceptions of stakeholders is also crucial going forward, and in our opinion such assessments could complement the development of administrative ‘indicators’ ^47^ to appraise where effort in post-award management is concentrated, assess its end value to research, and identify areas for further improvement.

### Recommendations for funders and institutions

We have drawn on the evidence of common issues and potential solutions in post-award management ( Table 6) to inform key recommendations for funders and HEIs. We believe these recommendations will be relevant to many funders internationally and could facilitate effective future changes to reduce unnecessary effort in research ^14^ or identify where more research is needed to inform feasible opportunities for improvement in post-award management.

### Recommendations for funders

• Funders should aim to simplify and harmonise their practices for monitoring and evaluation, ensuring they continue to collaborate, follow evidence of best practice, and attempt to overcome difficulties – such as being able to accommodate organisational differences (e.g., in priorities and monitoring requirements).
• Funders should evaluate and improve the frameworks they use for RIA but consider the effort involved for researchers and evaluators when changing monitoring and reporting requirements.
• Funders should ensure they engage HEIs, researchers, RMA experts, and any other relevant research stakeholders and sponsors, when making future decisions on practices that may impact research activity (such as adding reporting requirements).
• Funders should make sure they clarify for researchers the purpose of post-award information requirements, what happens to the data and who uses it, as well what data (e.g., related to impact) is relevant to report.
• Funders should ask researchers and HEIs for feedback on their programmes as part of routine reporting activity and include in this the time spent on administrative activities as a ‘metric’ of effort or burden.
• Funders should streamline their compliance and assurance requirements to reduce duplication in HEI processes and delay to the start of research – for instance, by adopting a principle of ‘ask once’ when requesting information from HEIs.

### Recommendations for HEIs

• HEIs should strengthen their grant management capacities, recognise the importance of non-research personnel in assisting with research operations, and particularly the role of research managers and administrators in enhancing the quality and success of research.
• More HEIs should make use of existing networks (such as the Association for Research Managers and Administrators) to share information and resources on effective award management. This could help towards harmonising HEI practices, such as through the use of more standardised processes (e.g., the Lambert agreement for research contracts) and common information management systems.
• HEIs should provide regular feedback to funders to drive continuous improvement in post-award management.

### Recommendations for both funders and HEIs

• Funders and HEIs should work with relevant suppliers (such as Jisc) to further improve interoperability between digital systems to better share information and reduce duplication in data management and reporting.
• Funders and HEIs should work with relevant organisations (such as euroCRIS) to improve transferability of grant-linked data between HEI and external systems, and promote application of universal standards to validate data.
• Funders and HEIs should both better support researchers through the post-award phase and with reporting activities, helping them build better working relationships, and secure future funding through the ultimate success of their research.

### Limitations

The broad nature of the topic and the breadth of terminology used to describe post-award management in the literature made screening for relevant papers more challenging than initially anticipated. Having to also manage a large number of citations and employ strict criteria for eligible literature, it is therefore possible that some relevant articles and resources will have been missed. Nevertheless, the literature sample we obtained contained more research data than anticipated and provides the key information to appraise the landscape of post-award management. We attempted to capture current funder practices as accurately as possible but acknowledge that, our funder sample is small and predominantly in the biomedical/health space, and second, and that funders’ websites will not necessarily capture every operational nuance and policy regarding their post-award practices. It also needs mentioning that research practices are constantly changing, and there is therefore a limit to how much current detail on practices can be obtained without consulting funders or HEIs themselves (for instance, as done in Ref. 78). Finally, we note that the findings of the review and especially any assessments of ‘effort’, should not be seen as reflective of all real-life experiences in research; the perspectives of researchers on funding and post-award management will vary and we have recently shown this when interviewing researchers in the UK. ^61^

## Conclusions

The overall need to manage and report on research is clear and widely appreciated. However, the effort can be considerable and reports where it is perceived as unnecessary need the support of more rigorous evidence, and consultations between researchers, HEIs, funders and other relevant stakeholders, so that key administrative barriers to efficient research delivery can be identified and addressed more collaboratively in a connected, interoperable research environment. In the meantime, HEIs and researchers could benefit from more administrative support services, and researchers could particularly benefit from guidance on ‘impact’ and training in post-award management. Funders could also find ways of reducing duplication and research waste in reporting, with a goal to minimise the effort required to report whilst increasing its value and accountability to research end-users.

## Data Availability

No data are associated with this article.
