# Supplementary material for: The post-award effort of managing and reporting on funded research: a scoping review
Source: F1000Res. 2023 Sep 28;12:863. Originally published 2023 Jul 20. [Version 2] doi: 10.12688/f1000research.133263.2 (PMC10570692; doi:10.12688/f1000research.133263.2)
Supplement: Peer review history

# Reviewer report: Reviewer response for version 2

**DOI:** 10.5256/f1000research.155674.r210649

**Published:** 2023-10-12

**Authors:** Simon Kerridge

**Affiliations:** 1 Kerridge Research Consulting, and (Honorary) Research & Innovation Services, University of Kent, Canterbury, England, UK

The authors have addressed my concerns admirably.

Are the rationale for, and objectives of, the Systematic Review clearly stated?

Yes

Is the statistical analysis and its interpretation appropriate?

Not applicable

Are sufficient details of the methods and analysis provided to allow replication by others?

Yes

Are the conclusions drawn adequately supported by the results presented in the review?

Yes

Reviewer Expertise:

Research Management and Administration

I confirm that I have read this submission and believe that I have an appropriate level of expertise to confirm that it is of an acceptable scientific standard.

# Reviewer report: Reviewer response for version 1

**DOI:** 10.5256/f1000research.146242.r194311

**Published:** 2023-08-23

**Authors:** Simon Kerridge

**Affiliations:** 1 Kerridge Research Consulting, and (Honorary) Research & Innovation Services, University of Kent, Canterbury, England, UK

Overall the article is thorough and very useful being an under-researched sub-area (post-award) in the under-researched area of research management and administration (RMA).

 However, there are a number of possible issues.

 One overarching limitation is the skew/focus on RMA in the biomedical arena, which is not made sufficiently clear.  Table 2 for example lists of the funder websites searched, and the databases used in the initial phase are predominantly biomedical.  This is not specifically an issue, but should be highlighted in the limitations / scope.

 Given the UK base of the authors it would perhaps have been sensible to have looked at the ARMA Professional Development Framework, which would have supported their scoping of the duties in post-award RMA in Table 4.

 One other issue was the inclusion of the Tickell report twice (the draft and the final), which may have skewed the data slightly.

 Please note that while these issues mean that the paper is not perfect, it is still an excellent piece of work and contributes greatly to the field, giving much practical guidance on how post-award RMA can be improved from across the ecosystem.

 In terms of the recommendations, the first for funders to harmonise might have mentioned some of the difficulties here - there have been many initiatives at RCUK (now UKRI) which have progressed... slowly.

 The third funder bullet talks about engagement with "HEIs, researchers, and other institutions and sponsors", given the general tenet of the article I would have expected to see RMAs listed here too.

 The second bullet for Recommendations for HEIs talks about forming networks for information sharing, it could be argued that ARMA fills this need, and has a post-award special interest group.  On the same point "standard contract agreement" might have discussed the long standing Lambert agreement.

 Recommendations for both - the second point talks about standards, and might have brought in euroCRIS.

 Finally, it would have been great to have seen the underlying working data referenced to aid transparency.

 There are a number of minor points:

- Given the international nature of the review it is not clear why RA is not listed in the "List of abbreviations" [doing so might aid discoverability in North America].  And then perhaps as both RA and RMA are used, it might have been useful to relate them to each other.
- One quote on p.23 is not in italics.
- On p.28 the first bullet under Hunter et al. has "( s" which should probably be "(s"
- On p.37 it was not clear to me why it was thought that there might be a "clear relationship between organisation size"...
- On p.38 at the top, "research sectors" was not clear to me ... just "research" ?
- Also on p.38 "Impact literacy" was introduced, but I was surprised not see a citation here - perhaps to Julie Bayley's work, e.g. https://books.emeraldinsight.com/book/detail/creating-meaningful-impact/?k=9781804551929 

However, as stated above, these are all fairly minor considerations and the overall content is rich and compelling and far outweighs these comments.

Are the rationale for, and objectives of, the Systematic Review clearly stated?

Yes

Is the statistical analysis and its interpretation appropriate?

Not applicable

Are sufficient details of the methods and analysis provided to allow replication by others?

Yes

Are the conclusions drawn adequately supported by the results presented in the review?

Yes

Reviewer Expertise:

Research Management and Administration

I confirm that I have read this submission and believe that I have an appropriate level of expertise to confirm that it is of an acceptable scientific standard, however I have significant reservations, as outlined above.

# Reviewer report: Reviewer response for version 1

**DOI:** 10.5256/f1000research.146242.r188988

**Published:** 2023-07-28

**Authors:** Caitlin Brandenburg

**Affiliations:** 1 Gold Coast Health, Parkwood, QLD, Australia; 2 Metro South Health, Eight Mile Plains, Qld, Australia

This scoping review on post-award reporting is interesting, robust, timely, and for the most part well-written. Methods were well-detailed and followed JBI scoping review standards. I have specific comments to strengthen the writing as outlined below.

 Abstract:

- “Inconsistent approaches to reporting and post-award management, and a growing demand for research information, can lead to perception of unnecessary administrative effort that impacts on decision-making and research activity”- meaning could be clearer, perhaps separate into 2 sentences?

Introduction:

- “involves reporting to funders on award-related activities,”- more specificity on who is reporting may help further define this process, for example “involves research grant recipients reporting…” or is the definition broader than that?
- “involves reporting to funders on award-related activities, and is how funders oversee the progress, outputs, outcomes, and impacts of the research they fund”- long sentence, would be best split into 2, with the second “This process is how funders oversee…”
- “The effort involved can be considerable..” as above could be more specific about who the effort is expended by - researchers?
- “feeling of ‘pressure’ among early career researchers”- why early career specifically? Is it that they are doing the bulk of this reporting work? Please elucidate.
- “An additional aim was to compare how funders currently approach post-award management, identify any unnecessary effort or need for improvement, and to use evidence of previous solutions to inform recommendations for both funders and HEIs.” Suggest use of semicolons here for consistency, as used in the list in the previous sentence.

Methods:

- “the broad definition” - should be “a broad definition”
- “refers to any process – funder or HEI – “ - the meaning here is unclear, as the structure of the sentence implies that funder/HEI are examples of a process. Please restructure.
- “and duplicates removed” - how were duplicates identified and removed? E.g. manual deduplication, use of EndNote duplication feature?
- Table 6 is too lengthy to be useful- please provide a further summary of the main themes in the main text for the ease of the reader.

Are the rationale for, and objectives of, the Systematic Review clearly stated?

Yes

Is the statistical analysis and its interpretation appropriate?

Not applicable

Are sufficient details of the methods and analysis provided to allow replication by others?

Yes

Are the conclusions drawn adequately supported by the results presented in the review?

Yes

Reviewer Expertise:

Meta-research

I confirm that I have read this submission and believe that I have an appropriate level of expertise to confirm that it is of an acceptable scientific standard.
